# Supplementary material for: Structure of human ORP3 ORD reveals conservation of a key function and ligand specificity in OSBP-related proteins
Source: PLoS One. 2021 Apr 15;16(4):e0248781. doi: 10.1371/journal.pone.0248781 (PMC8049286; doi:10.1371/journal.pone.0248781)
Supplement: S1 File — (PDF) [file pone.0248781.s001.pdf]

Preliminary Full wwPDB X-ray Structure Validation Report (i)

Nov 4, 2020 – 11:18 AM JST

Deposition ID : D_1300019243

This is a Preliminary Full wwPDB X-ray Structure Validation Report.

This report is produced by the wwPDB Deposition System during initial deposition but before annotation of the structure.

We welcome your comments at *validation@mail.wwpdb.org*
A user guide is available at
https://www.wwpdb.org/validation/2017/XrayValidationReportHelp
with specific help available everywhere you see the (i) symbol.

PRELIMINARY VALIDATION REPORT

The following versions of software and data (see references (i)) were used in the production of this report:

MolProbity	:	4.02b-467
Mogul	:	1.8.5 (274361), CSD as541be (2020)
Xtriage (Phenix)	:	1.13
EDS	:	2.14.6
buster-report	:	1.1.7 (2018)
Percentile statistics	:	20191225.v01 (using entries in the PDB archive December 25th 2019)
Refmac	:	5.8.0158
CCP4	:	7.0.044 (Gargrove)
Ideal geometry (proteins)	:	Engh & Huber (2001)
Ideal geometry (DNA, RNA)	:	Parkinson et al. (1996)
Validation Pipeline (wwPDB-VP)	:	2.14.6

1 Overall quality at a glance (i)

The following experimental techniques were used to determine the structure:
X-RAY DIFFRACTION

The reported resolution of this entry is 2.60 Å.

Percentile scores (ranging between 0-100) for global validation metrics of the entry are shown in the following graphic. The table shows the number of entries on which the scores are based.

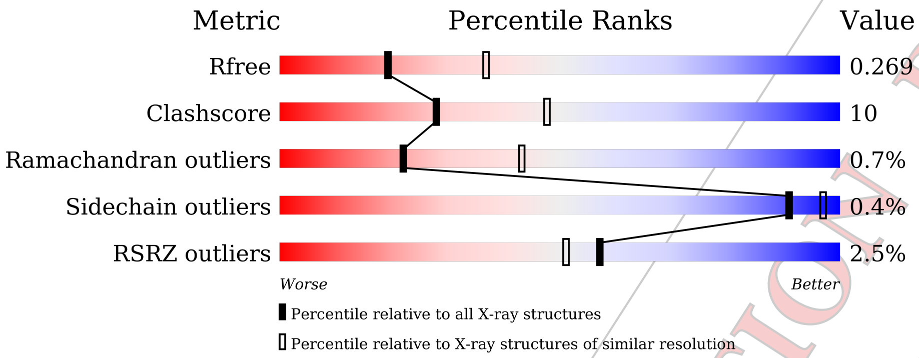

Metric	Whole archive (#Entries)	Similar resolution (#Entries, resolution range(Å))
R_{free}	130704	3163 (2.60-2.60)
Clashscore	141614	3518 (2.60-2.60)
Ramachandran outliers	138981	3455 (2.60-2.60)
Sidechain outliers	138945	3455 (2.60-2.60)
RSRZ outliers	127900	3104 (2.60-2.60)

The table below summarises the geometric issues observed across the polymeric chains and their fit to the electron density. The red, orange, yellow and green segments on the lower bar indicate the fraction of residues that contain outliers for >=3, 2, 1 and 0 types of geometric quality criteria respectively. A grey segment represents the fraction of residues that are not modelled. The numeric value for each fraction is indicated below the corresponding segment, with a dot representing fractions <=5% The upper red bar (where present) indicates the fraction of residues that have poor fit to the electron density. The numeric value is given above the bar.

Mol	Chain	Length	Quality of chain
1	A	376	3% / 77% 22% •
2	B	377	2% / 76% 21% •

PRELIMINARY VALIDATION REPORT

2 Entry composition (i)

There are 4 unique types of molecules in this entry. The entry contains 6309 atoms, of which 0 are hydrogens and 0 are deuteriums.

In the tables below, the ZeroOcc column contains the number of atoms modelled with zero occupancy, the AltConf column contains the number of residues with at least one atom in alternate conformation and the Trace column contains the number of residues modelled with at most 2 atoms.

- Molecule 1 is a protein.

Mol	Chain	Residues	Atoms					ZeroOcc	AltConf	Trace
			Total	C	N	O	S			
1	A	376	3083	1961	544	565	13	0	0	0

- Molecule 2 is a protein.

Mol	Chain	Residues	Atoms					ZeroOcc	AltConf	Trace
			Total	C	N	O	S			
2	B	377	3089	1964	545	567	13	0	0	0

- Molecule 3 is (2R)-3-{[(S)-hydroxy{[(1R,2R,3R,4R,5S,6R)-2,3,5,6-tetrahydroxy-4-(phosphonooxy)cyclohexyl]oxy}phosphoryl]oxy}propane-1,2-diyl dioctanoate (three-letter code: PIF) (formula: $C_{25}H_{48}O_{16}P_2$).

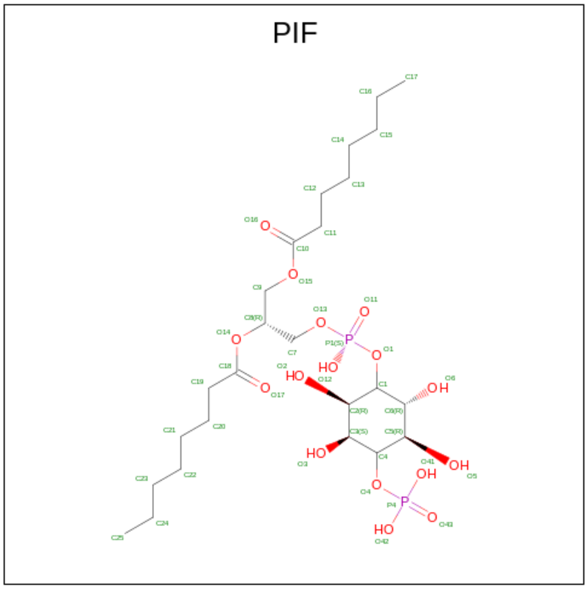

Mol	Chain	Residues	Atoms				ZeroOcc	AltConf
			Total	C	O	P		
3	A	1	43	25	16	2	0	0

Continued on next page...

PRELIMINARY VALIDATION REPORT

Continued from previous page...

Mol	Chain	Residues	Atoms				ZeroOcc	AltConf
			Total	C	O	P		
3	B	1	43	25	16	2	0	0

- Molecule 4 is water.

Mol	Chain	Residues	Atoms		ZeroOcc	AltConf
			Total	O		
4	S	51	51	51	0	0

PRELIMINARY VALIDATION REPORT

3 Residue-property plots (i)

These plots are drawn for all protein, RNA, DNA and oligosaccharide chains in the entry. The first graphic for a chain summarises the proportions of the various outlier classes displayed in the second graphic. The second graphic shows the sequence view annotated by issues in geometry and electron density. Residues are color-coded according to the number of geometric quality criteria for which they contain at least one outlier: green = 0, yellow = 1, orange = 2 and red = 3 or more. A red dot above a residue indicates a poor fit to the electron density (RSRZ > 2). Stretches of 2 or more consecutive residues without any outlier are shown as a green connector. Residues present in the sample, but not in the model, are shown in grey.

- Molecule 1:

Chain A:

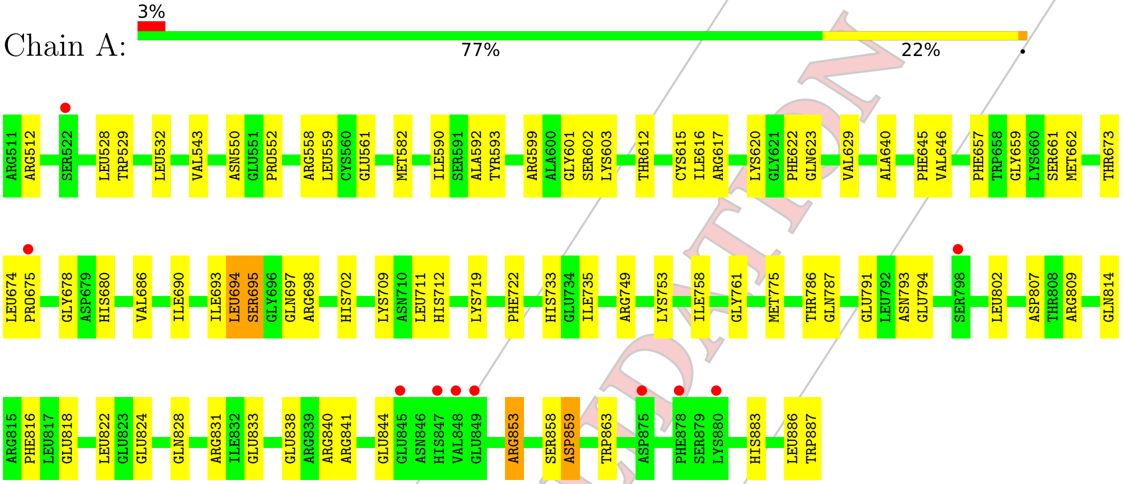

- Molecule 2:

Chain B:

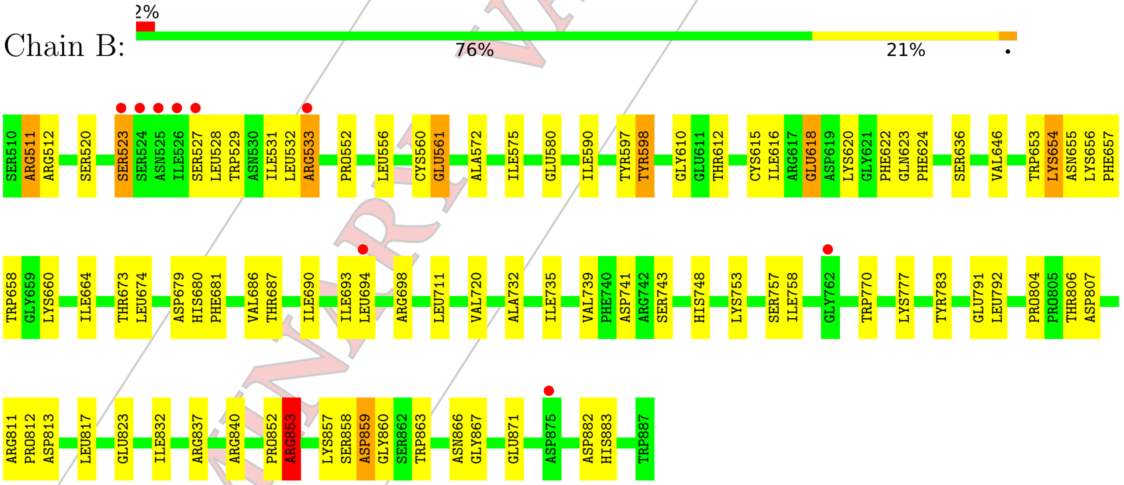

PRELIMINARY VALIDATION REPORT

4 Data and refinement statistics (i)

Property	Value	Source
Space group	P 31 2 1	Depositor
Cell constants<br>a, b, c, α, β, γ	95.80Å 95.80Å 190.46Å<br>90.00° 90.00° 120.00°	Depositor
Resolution (Å)	46.45 – 2.60<br>46.45 – 2.60	Depositor<br>EDS
% Data completeness<br>(in resolution range)	99.4 (46.45-2.60)<br>99.4 (46.45-2.60)	Depositor<br>EDS
R_{merge}	(Not available)	Depositor
R_{sym}	(Not available)	Depositor
$< I/\sigma(I) >$ [1]	5.12 (at 2.61Å)	Xtriage
Refinement program	phenix.refine 1.18.2_3874, PHENIX 1.18.2_3874	Depositor
R, R_{free}	0.220 , 0.269<br>0.220 , 0.269	Depositor<br>DCC
R_{free} test set	2013 reflections (6.38%)	wwPDB-VP
Wilson B-factor (Å^2)	57.5	Xtriage
Anisotropy	0.275	Xtriage
Bulk solvent k_{sol}(e/Å^3), B_{sol}(Å^2)	0.34 , 41.3	EDS
L-test for twinning[2]	$< \|L\| > = 0.50$, $< L^2 > = 0.34$	Xtriage
Estimated twinning fraction	0.025 for -h,-k,l	Xtriage
F_o,F_c correlation	0.94	EDS
Total number of atoms	6309	wwPDB-VP
Average B, all atoms (Å^2)	61.0	wwPDB-VP

Xtriage's analysis on translational NCS is as follows: *The largest off-origin peak in the Patterson function is 3.39% of the height of the origin peak. No significant pseudotranslation is detected.*

[1] Intensities estimated from amplitudes.

[2] Theoretical values of $< |L| >$, $< L^2 >$ for acentric reflections are 0.5, 0.333 respectively for untwinned datasets, and 0.375, 0.2 for perfectly twinned datasets.

PRELIMINARY VALIDATION REPORT

5 Model quality (i)

5.1 Standard geometry (i)

Bond lengths and bond angles in the following residue types are not validated in this section: PIF

The Z score for a bond length (or angle) is the number of standard deviations the observed value is removed from the expected value. A bond length (or angle) with $|Z| > 5$ is considered an outlier worth inspection. RMSZ is the root-mean-square of all Z scores of the bond lengths (or angles).

Mol	Chain	Bond lengths		Bond angles	
		RMSZ	$\#\|Z\| >5$	RMSZ	$\#\|Z\| >5$
1	A	0.52	0/3173	0.81	5/4298 (0.1%)
2	B	0.64	7/3179 (0.2%)	0.88	11/4306 (0.3%)
All	All	0.58	7/6352 (0.1%)	0.84	16/8604 (0.2%)

Chiral center outliers are detected by calculating the chiral volume of a chiral center and verifying if the center is modelled as a planar moiety or with the opposite hand.A planarity outlier is detected by checking planarity of atoms in a peptide group, atoms in a mainchain group or atoms of a sidechain that are expected to be planar.

Mol	Chain	#Chirality outliers	#Planarity outliers
2	B	0	1

All (7) bond length outliers are listed below:

Mol	Chain	Res	Type	Atoms	Z	Observed(Å)	Ideal(Å)
2	B	823	GLU	CD-OE2	10.25	1.36	1.25
2	B	654	LYS	CE-NZ	5.94	1.63	1.49
2	B	654	LYS	CA-CB	5.84	1.66	1.53
2	B	618	GLU	CG-CD	5.79	1.60	1.51
2	B	618	GLU	CD-OE1	5.24	1.31	1.25
2	B	618	GLU	CD-OE2	5.22	1.31	1.25
2	B	561	GLU	CD-OE2	5.05	1.31	1.25

All (16) bond angle outliers are listed below:

Mol	Chain	Res	Type	Atoms	Z	Observed(^{o})	Ideal(^{o})
2	B	533	ARG	NE-CZ-NH2	-10.29	115.15	120.30
2	B	654	LYS	CB-CG-CD	8.32	133.25	111.60
2	B	853	ARG	NE-CZ-NH1	-8.22	116.19	120.30
1	A	695	SER	C-N-CA	8.01	139.12	122.30

Continued on next page...

PRELIMINARY VALIDATION REPORT

Continued from previous page...

Mol	Chain	Res	Type	Atoms	Z	Observed(^{o})	Ideal(^{o})
2	B	654	LYS	N-CA-C	-7.83	89.85	111.00
2	B	523	SER	N-CA-C	6.82	129.43	111.00
1	A	694	LEU	CA-CB-CG	-5.81	101.94	115.30
1	A	853	ARG	NE-CZ-NH1	-5.79	117.41	120.30
2	B	853	ARG	CA-CB-CG	5.60	125.73	113.40
1	A	802	LEU	CB-CG-CD2	-5.45	101.73	111.00
2	B	533	ARG	CG-CD-NE	-5.44	100.38	111.80
2	B	528	LEU	CB-CG-CD2	-5.42	101.78	111.00
2	B	857	LYS	CB-CG-CD	-5.35	97.70	111.60
1	A	695	SER	N-CA-C	5.29	125.29	111.00
2	B	866	ASN	C-N-CA	5.08	132.97	122.30
2	B	511	ARG	CB-CG-CD	-5.08	98.40	111.60

There are no chirality outliers.

All (1) planarity outliers are listed below:

Mol	Chain	Res	Type	Group
2	B	561	GLU	Sidechain

5.2 Too-close contacts (i)

In the following table, the Non-H and H(model) columns list the number of non-hydrogen atoms and hydrogen atoms in the chain respectively. The H(added) column lists the number of hydrogen atoms added and optimized by MolProbity. The Clashes column lists the number of clashes within the asymmetric unit, whereas Symm-Clashes lists symmetry related clashes.

Mol	Chain	Non-H	H(model)	H(added)	Clashes	Symm-Clashes
1	A	3083	0	2972	63	1
2	B	3089	0	2977	59	2
3	A	43	0	45	5	0
3	B	43	0	45	0	0
4	S	51	0	0	10	0
All	All	6309	0	6039	122	2

The all-atom clashscore is defined as the number of clashes found per 1000 atoms (including hydrogen atoms). The all-atom clashscore for this structure is 10.

All (122) close contacts within the same asymmetric unit are listed below, sorted by their clash magnitude.

PRELIMINARY VALIDATION REPORT

Atom-1	Atom-2	Interatomic distance (Å)	Clash overlap (Å)
1:A:809:ARG:HD2	4:S:46:HOH:O	1.75	0.85
1:A:601:GLY:HA3	1:A:698:ARG:HD3	1.60	0.84
1:A:615:CYS:SG	4:S:28:HOH:O	2.34	0.83
1:A:807:ASP:OD2	4:S:46:HOH:O	1.96	0.83
1:A:693:ILE:HD12	1:A:693:ILE:H	1.51	0.75
1:A:662:MET:HE1	3:A:902:PIF:H23A	1.67	0.75
2:B:590:ILE:HD13	2:B:735:ILE:HD13	1.70	0.74
1:A:620:LYS:HD3	1:A:622:PHE:HE1	1.53	0.72
2:B:658:TRP:O	4:S:29:HOH:O	2.09	0.69
2:B:660:LYS:HA	2:B:694:LEU:HD23	1.75	0.68
2:B:735:ILE:HD11	2:B:758:ILE:HD12	1.74	0.68
1:A:552:PRO:HB3	1:A:818:GLU:HG2	1.76	0.67
2:B:686:VAL:O	4:S:20:HOH:O	2.13	0.66
1:A:719:LYS:NZ	4:S:36:HOH:O	2.27	0.66
2:B:512:ARG:HB3	2:B:791:GLU:HG2	1.78	0.65
2:B:532:LEU:HB3	2:B:657:PHE:CE1	2.31	0.65
2:B:735:ILE:O	2:B:735:ILE:HG13	1.98	0.63
1:A:853:ARG:NH1	1:A:883:HIS:HD2	1.97	0.63
2:B:529:TRP:CE2	2:B:694:LEU:HD12	2.35	0.61
2:B:811:ARG:HG2	2:B:813:ASP:OD1	2.00	0.60
1:A:844:GLU:OE1	1:A:844:GLU:HA	2.02	0.59
1:A:853:ARG:NH1	1:A:883:HIS:CD2	2.71	0.59
2:B:646:VAL:HB	2:B:673:THR:OG1	2.03	0.59
1:A:853:ARG:HH12	1:A:883:HIS:CD2	2.21	0.59
2:B:590:ILE:HD11	2:B:720:VAL:HG11	1.86	0.58
1:A:833:GLU:HG2	3:A:902:PIF:H5	1.83	0.58
1:A:678:GLY:O	1:A:712:HIS:ND1	2.28	0.58
2:B:660:LYS:N	4:S:29:HOH:O	2.25	0.57
1:A:793:ASN:HB3	1:A:818:GLU:OE2	2.05	0.57
1:A:561:GLU:CD	1:A:599:ARG:HH12	2.08	0.56
2:B:693:ILE:O	2:B:694:LEU:HB2	2.05	0.56
2:B:871:GLU:OE2	2:B:871:GLU:N	2.39	0.56
2:B:572:ALA:HA	2:B:575:ILE:HD12	1.88	0.56
2:B:741:ASP:OD1	2:B:743:SER:OG	2.17	0.56
1:A:550:ASN:OD1	1:A:603:LYS:HD3	2.07	0.55
1:A:840:ARG:CZ	1:A:840:ARG:HB3	2.36	0.55
1:A:646:VAL:HB	1:A:673:THR:OG1	2.07	0.55
2:B:664:ILE:HD12	2:B:690:ILE:HD11	1.88	0.54
2:B:653:TRP:HB3	2:B:655:ASN:OD1	2.08	0.53
1:A:593:TYR:OH	1:A:686:VAL:HG12	2.08	0.53
2:B:612:THR:OG1	2:B:806:THR:HG23	2.09	0.53
2:B:590:ILE:HD13	2:B:735:ILE:CD1	2.38	0.53

Continued on next page...

PRELIMINARY VALIDATION REPORT

Continued from previous page...

Atom-1	Atom-2	Interatomic distance (Å)	Clash overlap (Å)
1:A:629:VAL:HG21	1:A:863:TRP:CD1	2.44	0.52
2:B:777:LYS:HD3	2:B:777:LYS:C	2.29	0.52
1:A:735:ILE:HD11	1:A:758:ILE:HD12	1.92	0.52
2:B:693:ILE:HA	2:B:698:ARG:HE	1.75	0.51
2:B:837:ARG:HG2	2:B:840:ARG:HH12	1.73	0.51
1:A:838:GLU:O	1:A:841:ARG:HB3	2.09	0.51
2:B:757:SER:O	2:B:758:ILE:HD13	2.10	0.51
1:A:661:SER:HA	1:A:693:ILE:HD11	1.91	0.51
1:A:645:PHE:HA	1:A:675:PRO:HD3	1.93	0.51
1:A:678:GLY:C	1:A:712:HIS:HD1	2.12	0.50
2:B:693:ILE:HA	2:B:698:ARG:NE	2.27	0.50
2:B:616:ILE:HG23	2:B:623:GLN:HG2	1.94	0.50
1:A:552:PRO:O	1:A:814:GLN:NE2	2.46	0.49
1:A:775:MET:SD	1:A:786:THR:HG22	2.53	0.49
1:A:612:THR:O	1:A:807:ASP:HA	2.13	0.49
2:B:758:ILE:HG12	2:B:770:TRP:CE3	2.48	0.49
1:A:543:VAL:O	3:A:902:PIF:H7A	2.13	0.49
2:B:674:LEU:HB2	2:B:679:ASP:HB2	1.95	0.48
2:B:882:ASP:OD1	2:B:883:HIS:N	2.46	0.48
1:A:532:LEU:HB3	1:A:657:PHE:CD2	2.47	0.48
2:B:620:LYS:HD3	2:B:622:PHE:HE2	1.78	0.48
1:A:690:ILE:O	1:A:693:ILE:HD11	2.13	0.48
2:B:804:PRO:HB2	2:B:806:THR:HG22	1.95	0.48
2:B:758:ILE:HG12	2:B:770:TRP:HE3	1.79	0.48
1:A:709:LYS:NZ	4:S:47:HOH:O	2.37	0.47
2:B:837:ARG:HG2	2:B:840:ARG:NH1	2.29	0.47
2:B:527:SER:O	2:B:531:ILE:HG12	2.13	0.47
1:A:623:GLN:O	1:A:640:ALA:HA	2.14	0.47
2:B:610:GLY:O	2:B:853:ARG:NH1	2.47	0.47
2:B:732:ALA:O	2:B:753:LYS:HE3	2.15	0.47
2:B:597:TYR:HD2	2:B:598:TYR:CE2	2.33	0.47
1:A:697:GLN:O	1:A:697:GLN:HG2	2.15	0.47
1:A:702:HIS:O	1:A:722:PHE:HB3	2.15	0.47
2:B:806:THR:HG21	2:B:853:ARG:HH22	1.81	0.46
1:A:512:ARG:NH1	1:A:617:ARG:HD2	2.30	0.46
2:B:858:SER:O	2:B:859:ASP:C	2.53	0.46
1:A:603:LYS:HE2	3:A:902:PIF:O14	2.16	0.46
1:A:680:HIS:CG	1:A:711:LEU:HD12	2.51	0.46
1:A:694:LEU:HA	1:A:694:LEU:HD23	1.48	0.46
1:A:620:LYS:HB2	1:A:622:PHE:CD1	2.50	0.45
2:B:615:CYS:SG	4:S:1:HOH:O	2.43	0.45

Continued on next page...

PRELIMINARY VALIDATION REPORT

Continued from previous page...

Atom-1	Atom-2	Interatomic distance (Å)	Clash overlap (Å)
1:A:558:ARG:NH1	1:A:602:SER:OG	2.49	0.45
1:A:529:TRP:CZ3	1:A:694:LEU:HB3	2.52	0.45
2:B:618:GLU:OE1	2:B:618:GLU:HA	2.16	0.45
2:B:575:ILE:HG21	2:B:580:GLU:HB2	1.98	0.45
2:B:556:LEU:HD23	2:B:636:SER:HB3	1.99	0.44
2:B:735:ILE:CD1	2:B:758:ILE:HD12	2.42	0.44
2:B:739:VAL:HB	2:B:748:HIS:HB2	2.00	0.44
2:B:520:SER:HB3	2:B:783:TYR:H	1.82	0.44
2:B:560:CYS:HB2	2:B:624:PHE:CZ	2.53	0.44
2:B:679:ASP:HB3	2:B:681:PHE:CE1	2.52	0.44
1:A:674:LEU:HA	1:A:675:PRO:HD3	1.76	0.44
1:A:661:SER:HA	1:A:693:ILE:CD1	2.48	0.43
2:B:858:SER:O	2:B:860:GLY:N	2.50	0.43
2:B:686:VAL:HG22	2:B:687:THR:H	1.85	0.42
1:A:620:LYS:HB2	1:A:622:PHE:HD1	1.82	0.42
2:B:680:HIS:CD2	2:B:711:LEU:HD12	2.54	0.42
2:B:812:PRO:HD2	2:B:832:ILE:HD11	2.00	0.42
1:A:733:HIS:O	1:A:753:LYS:HA	2.19	0.42
1:A:592:ALA:HB2	4:S:2:HOH:O	2.19	0.42
1:A:822:LEU:HD23	1:A:822:LEU:HA	1.84	0.42
2:B:792:LEU:HD23	2:B:792:LEU:HA	1.79	0.42
1:A:886:LEU:HB2	1:A:887:TRP:CE3	2.55	0.42
1:A:794:GLU:O	1:A:809:ARG:NH1	2.53	0.42
2:B:852:PRO:HG2	2:B:863:TRP:CZ3	2.55	0.41
2:B:620:LYS:HD3	2:B:622:PHE:CE2	2.55	0.41
2:B:612:THR:O	2:B:807:ASP:HA	2.20	0.41
1:A:590:ILE:HD13	1:A:735:ILE:CD1	2.51	0.41
1:A:616:ILE:HD13	1:A:616:ILE:HA	1.91	0.41
1:A:858:SER:O	1:A:859:ASP:C	2.58	0.41
2:B:552:PRO:HG3	2:B:817:LEU:HD23	2.03	0.41
1:A:528:LEU:HD12	1:A:528:LEU:HA	1.90	0.41
1:A:749:ARG:HB2	1:A:761:GLY:O	2.21	0.41
2:B:655:ASN:O	2:B:656:LYS:HD2	2.21	0.41
1:A:828:GLN:OE1	1:A:831:ARG:NH1	2.54	0.41
1:A:816:PHE:CE2	1:A:824:GLU:HG3	2.56	0.40
1:A:582:MET:HE3	1:A:582:MET:O	2.21	0.40
1:A:559:LEU:HD23	1:A:559:LEU:HA	1.96	0.40
1:A:787:GLN:O	1:A:791:GLU:HG3	2.21	0.40
1:A:603:LYS:HB2	3:A:902:PIF:H22	2.03	0.40

All (2) symmetry-related close contacts are listed below. The label for Atom-2 includes the symmetry operator and encoded unit-cell translations to be applied.

PRELIMINARY VALIDATION REPORT

Atom-1	Atom-2	Interatomic distance (Å)	Clash overlap (Å)
2:B:533:ARG:NH2	2:B:867:GLY:O[4_545]	1.98	0.22
1:A:674:LEU:O	2:B:511:ARG:NH1[5_444]	2.11	0.09

5.3 Torsion angles (i)

5.3.1 Protein backbone (i)

In the following table, the Percentiles column shows the percent Ramachandran outliers of the chain as a percentile score with respect to all X-ray entries followed by that with respect to entries of similar resolution.

The Analysed column shows the number of residues for which the backbone conformation was analysed, and the total number of residues.

Mol	Chain	Analysed	Favoured	Allowed	Outliers	Percentiles	
1	A	374/376 (100%)	349 (93%)	22 (6%)	3 (1%)	19	39
2	B	375/377 (100%)	351 (94%)	22 (6%)	2 (0%)	29	52
All	All	749/753 (100%)	700 (94%)	44 (6%)	5 (1%)	22	43

All (5) Ramachandran outliers are listed below:

Mol	Chain	Res	Type
1	A	695	SER
2	B	859	ASP
1	A	659	GLY
1	A	859	ASP
2	B	523	SER

5.3.2 Protein sidechains (i)

In the following table, the Percentiles column shows the percent sidechain outliers of the chain as a percentile score with respect to all X-ray entries followed by that with respect to entries of similar resolution.

The Analysed column shows the number of residues for which the sidechain conformation was analysed, and the total number of residues.

Mol	Chain	Analysed	Rotameric	Outliers	Percentiles	
1	A	337/337 (100%)	337 (100%)	0	100	100
2	B	338/338 (100%)	335 (99%)	3 (1%)	78	91

Continued on next page...

PRELIMINARY VALIDATION REPORT

Continued from previous page...

Mol	Chain	Analysed	Rotameric	Outliers	Percentiles	
All	All	675/675 (100%)	672 (100%)	3 (0%)	91	97

All (3) residues with a non-rotameric sidechain are listed below:

Mol	Chain	Res	Type
2	B	598	TYR
2	B	654	LYS
2	B	853	ARG

Some sidechains can be flipped to improve hydrogen bonding and reduce clashes. All (3) such sidechains are listed below:

Mol	Chain	Res	Type
1	A	644	ASN
1	A	691	HIS
1	A	883	HIS

5.3.3 RNA (i)

There are no RNA molecules in this entry.

5.4 Non-standard residues in protein, DNA, RNA chains (i)

There are no non-standard protein/DNA/RNA residues in this entry.

5.5 Carbohydrates (i)

There are no monosaccharides in this entry.

5.6 Ligand geometry (i)

2 ligands are modelled in this entry.

In the following table, the Counts columns list the number of bonds (or angles) for which Mogul statistics could be retrieved, the number of bonds (or angles) that are observed in the model and the number of bonds (or angles) that are defined in the Chemical Component Dictionary. The Link column lists molecule types, if any, to which the group is linked. The Z score for a bond length (or angle) is the number of standard deviations the observed value is removed from the expected value. A bond length (or angle) with $|Z| > 2$ is considered an outlier worth inspection. RMSZ is the root-mean-square of all Z scores of the bond lengths (or angles).

PRELIMINARY VALIDATION REPORT

Mol	Type	Chain	Res	Link	Bond lengths			Bond angles		
					Counts	RMSZ	#|Z| > 2	Counts	RMSZ	#|Z| > 2
3	PIF	B	902	-	43,43,43	1.16	5 (11%)	55,58,58	1.28	7 (12%)
3	PIF	A	902	-	43,43,43	1.25	5 (11%)	55,58,58	1.16	5 (9%)

In the following table, the Chirals column lists the number of chiral outliers, the number of chiral centers analysed, the number of these observed in the model and the number defined in the Chemical Component Dictionary. Similar counts are reported in the Torsion and Rings columns. '-' means no outliers of that kind were identified.

Mol	Type	Chain	Res	Link	Chirals	Torsions	Rings
3	PIF	B	902	-	-	17/39/63/63	0/1/1/1
3	PIF	A	902	-	-	16/39/63/63	0/1/1/1

All (10) bond length outliers are listed below:

Mol	Chain	Res	Type	Atoms	Z	Observed(Å)	Ideal(Å)
3	A	902	PIF	P4-O4	3.42	1.65	1.59
3	B	902	PIF	P4-O4	3.19	1.65	1.59
3	A	902	PIF	O15-C10	2.99	1.42	1.33
3	A	902	PIF	O14-C18	2.77	1.42	1.34
3	B	902	PIF	O15-C10	2.48	1.40	1.33
3	A	902	PIF	P1-O13	2.20	1.68	1.59
3	A	902	PIF	C25-C24	-2.17	1.32	1.49
3	B	902	PIF	O15-C9	-2.14	1.40	1.45
3	B	902	PIF	C25-C24	-2.13	1.32	1.49
3	B	902	PIF	O14-C18	2.10	1.40	1.34

All (12) bond angle outliers are listed below:

Mol	Chain	Res	Type	Atoms	Z	Observed(^{o})	Ideal(^{o})
3	A	902	PIF	O14-C18-C19	3.61	119.28	111.50
3	B	902	PIF	O14-C18-C19	3.43	118.89	111.50
3	B	902	PIF	C9-C8-C7	-2.89	104.95	111.79
3	B	902	PIF	O15-C10-C11	2.85	120.85	111.91
3	A	902	PIF	C9-C8-C7	-2.74	105.30	111.79
3	B	902	PIF	C6-C5-C4	2.65	115.72	109.68
3	A	902	PIF	O15-C10-C11	2.58	119.99	111.91
3	B	902	PIF	O1-C1-C6	-2.52	102.81	108.66
3	A	902	PIF	C5-C6-C1	2.48	115.34	109.68
3	A	902	PIF	C8-O14-C18	2.18	123.15	117.79
3	B	902	PIF	C9-O15-C10	-2.12	109.27	117.12
3	B	902	PIF	O6-C6-C1	-2.08	104.42	109.94

PRELIMINARY VALIDATION REPORT

There are no chirality outliers.

All (33) torsion outliers are listed below:

Mol	Chain	Res	Type	Atoms
3	B	902	PIF	C19-C20-C21-C22
3	B	902	PIF	C18-C19-C20-C21
3	A	902	PIF	C18-C19-C20-C21
3	A	902	PIF	C12-C13-C14-C15
3	A	902	PIF	O17-C18-O14-C8
3	A	902	PIF	C19-C18-O14-C8
3	A	902	PIF	C11-C12-C13-C14
3	B	902	PIF	C12-C13-C14-C15
3	B	902	PIF	C11-C12-C13-C14
3	A	902	PIF	C14-C15-C16-C17
3	A	902	PIF	O13-C7-C8-O14
3	A	902	PIF	C20-C21-C22-C23
3	A	902	PIF	C13-C14-C15-C16
3	B	902	PIF	C7-C8-C9-O15
3	B	902	PIF	O13-C7-C8-O14
3	B	902	PIF	O14-C8-C9-O15
3	B	902	PIF	C2-C1-O1-P1
3	B	902	PIF	C6-C1-O1-P1
3	B	902	PIF	C20-C21-C22-C23
3	B	902	PIF	O13-C7-C8-C9
3	A	902	PIF	O13-C7-C8-C9
3	B	902	PIF	C22-C23-C24-C25
3	A	902	PIF	C10-C11-C12-C13
3	B	902	PIF	C7-O13-P1-O1
3	A	902	PIF	C7-O13-P1-O1
3	A	902	PIF	C2-C1-O1-P1
3	B	902	PIF	C13-C14-C15-C16
3	B	902	PIF	C21-C22-C23-C24
3	A	902	PIF	O14-C18-C19-C20
3	A	902	PIF	C7-O13-P1-O11
3	B	902	PIF	O14-C18-C19-C20
3	B	902	PIF	O17-C18-C19-C20
3	A	902	PIF	O17-C18-C19-C20

There are no ring outliers.

1 monomer is involved in 5 short contacts:

Mol	Chain	Res	Type	Clashes	Symm-Clashes
3	A	902	PIF	5	0

PRELIMINARY VALIDATION REPORT

The following is a two-dimensional graphical depiction of Mogul quality analysis of bond lengths, bond angles, torsion angles, and ring geometry for all instances of the Ligand of Interest. In addition, ligands with molecular weight > 250 and outliers as shown on the validation Tables will also be included. For torsion angles, if less then 5% of the Mogul distribution of torsion angles is within 10 degrees of the torsion angle in question, then that torsion angle is considered an outlier. Any bond that is central to one or more torsion angles identified as an outlier by Mogul will be highlighted in the graph. For rings, the root-mean-square deviation (RMSD) between the ring in question and similar rings identified by Mogul is calculated over all ring torsion angles. If the average RMSD is greater than 60 degrees and the minimal RMSD between the ring in question and any Mogul-identified rings is also greater than 60 degrees, then that ring is considered an outlier. The outliers are highlighted in purple. The color gray indicates Mogul did not find sufficient equivalents in the CSD to analyse the geometry.

Ligand PIF B 902

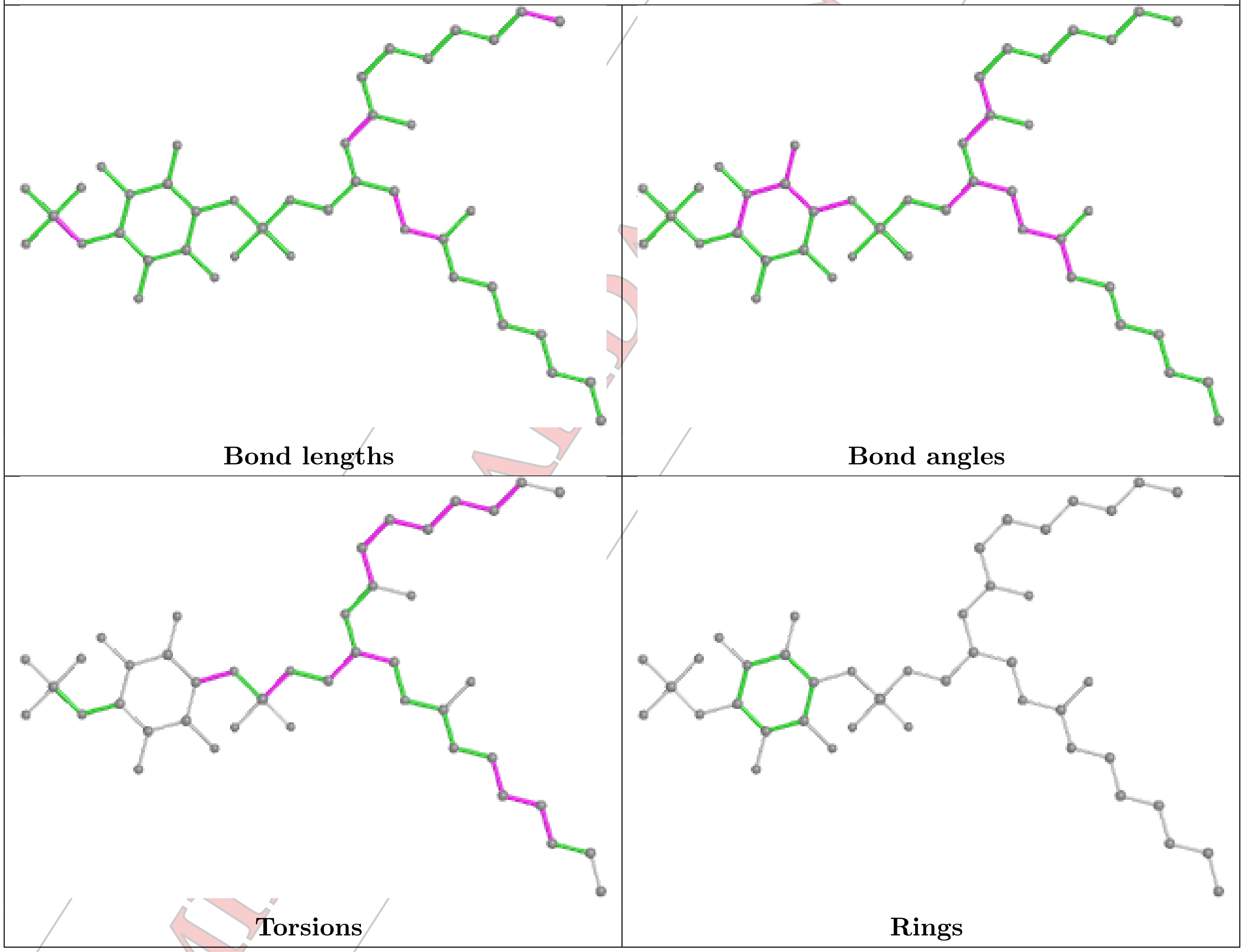

Ligand PIF A 902

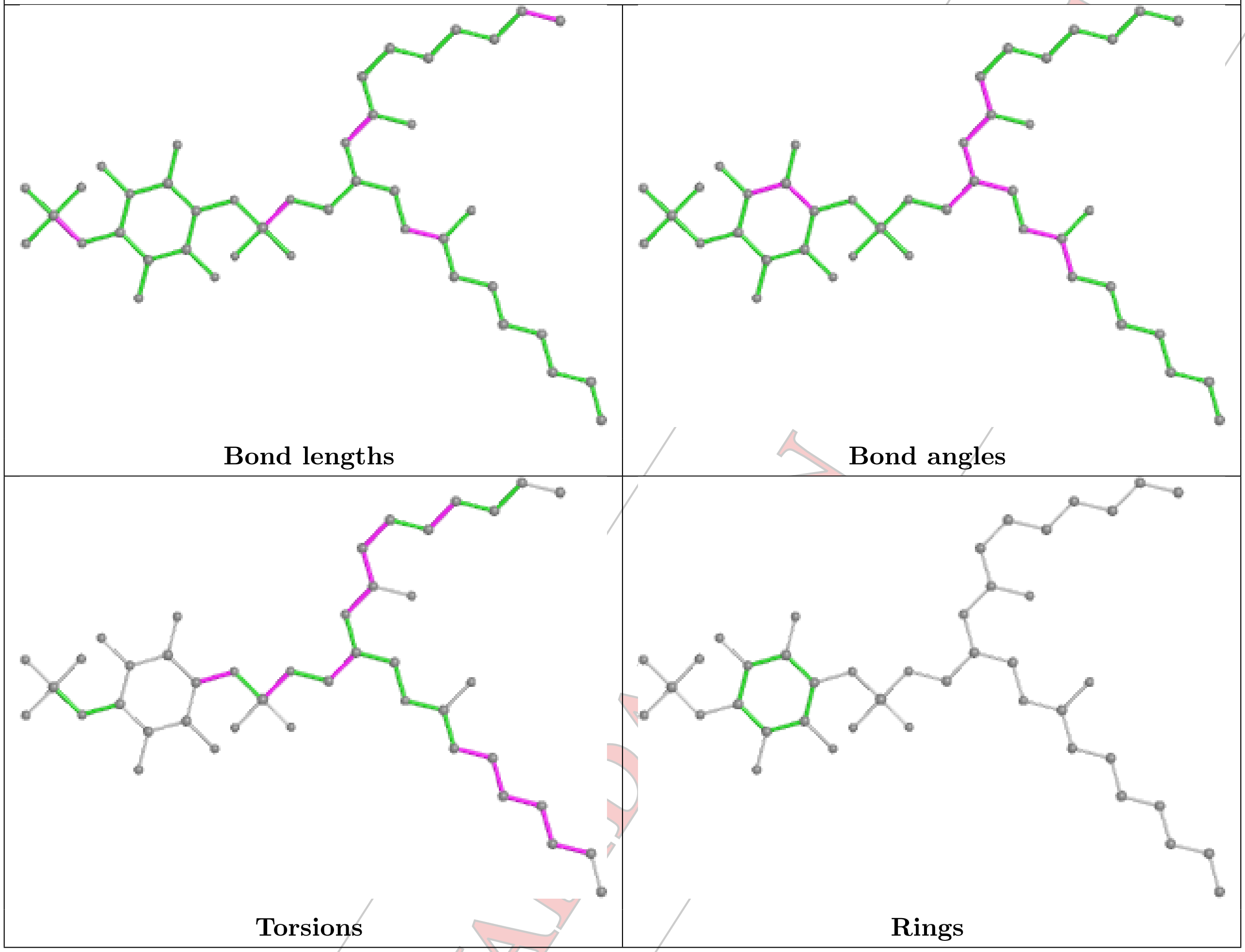

5.7 Other polymers (i)

There are no such residues in this entry.

5.8 Polymer linkage issues (i)

There are no chain breaks in this entry.

PRELIMINARY VALIDATION

6 Fit of model and data (i)

6.1 Protein, DNA and RNA chains (i)

In the following table, the column labelled ‘#RSRZ> 2’ contains the number (and percentage) of RSRZ outliers, followed by percent RSRZ outliers for the chain as percentile scores relative to all X-ray entries and entries of similar resolution. The OWAB column contains the minimum, median, 95th percentile and maximum values of the occupancy-weighted average B-factor per residue. The column labelled ‘Q< 0.9’ lists the number of (and percentage) of residues with an average occupancy less than 0.9.

Mol	Chain	Analysed	<RSRZ>	#RSRZ>2	OWAB($Å^2$)	Q<0.9
1	A	376/376 (100%)	0.01	10 (2%) 54 48	43, 64, 95, 109	0
2	B	377/377 (100%)	-0.15	9 (2%) 59 53	36, 52, 89, 127	0
All	All	753/753 (100%)	-0.07	19 (2%) 57 51	36, 59, 92, 127	0

All (19) RSRZ outliers are listed below:

Mol	Chain	Res	Type	RSRZ
2	B	526	ILE	9.8
1	A	675	PRO	3.8
1	A	847	HIS	3.8
1	A	798	SER	3.7
2	B	694	LEU	3.4
1	A	848	VAL	3.2
1	A	849	GLU	3.2
1	A	875	ASP	3.2
1	A	522	SER	3.2
2	B	527	SER	3.2
2	B	875	ASP	2.9
2	B	533	ARG	2.7
1	A	878	PHE	2.5
2	B	524	SER	2.4
1	A	880	LYS	2.4
2	B	762	GLY	2.3
2	B	525	ASN	2.2
1	A	845	GLU	2.2
2	B	523	SER	2.2

6.2 Non-standard residues in protein, DNA, RNA chains (i)

There are no non-standard protein/DNA/RNA residues in this entry.

PRELIMINARY VALIDATION REPORT

6.3 Carbohydrates (i)

There are no monosaccharides in this entry.

6.4 Ligands (i)

In the following table, the Atoms column lists the number of modelled atoms in the group and the number defined in the chemical component dictionary. The B-factors column lists the minimum, median, 95th percentile and maximum values of B factors of atoms in the group. The column labelled 'Q< 0.9' lists the number of atoms with occupancy less than 0.9.

Mol	Type	Chain	Res	Atoms	RSCC	RSR	B-factors(Å^2)	Q<0.9
3	PIF	B	902	43/?	0.97	0.18	44,60,81,91	0
3	PIF	A	902	43/?	0.97	0.15	52,61,69,73	0

The following is a graphical depiction of the model fit to experimental electron density of all instances of the Ligand of Interest. In addition, ligands with molecular weight > 250 and outliers as shown on the geometry validation Tables will also be included. Each fit is shown from different orientation to approximate a three-dimensional view.

Electron density around PIF B 902:

$2mF_o$-DF_c (at 0.7 rmsd) in gray
mF_o-DF_c (at 3 rmsd) in purple (negative)
and green (positive)

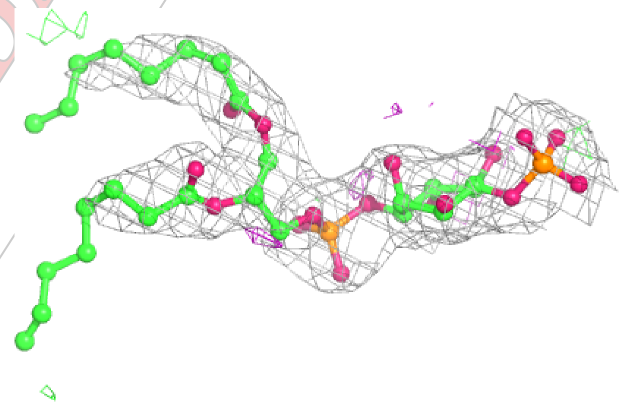

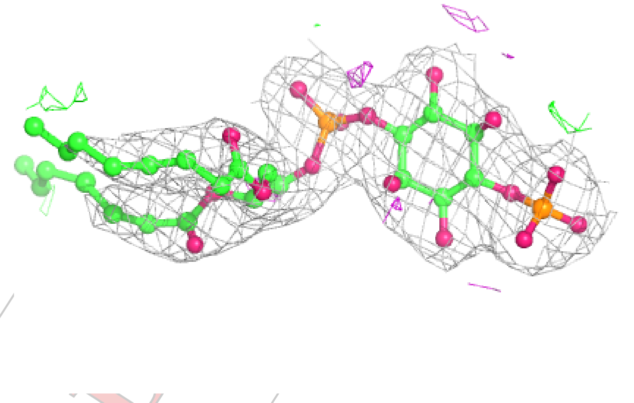

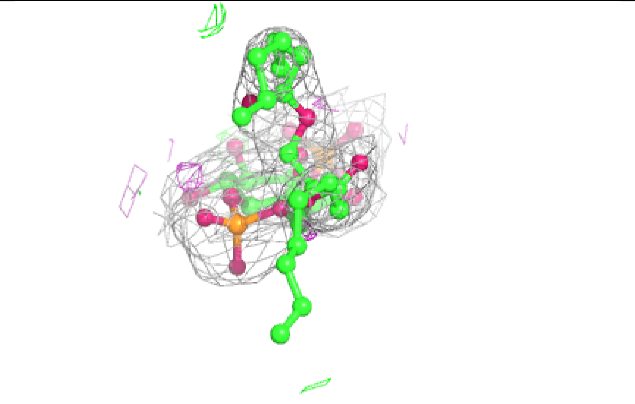

PRELIMINARY VALIDATION REPORT

Electron density around PIF A 902:

$2mF_o$-DF_c (at 0.7 rmsd) in gray
mF_o-DF_c (at 3 rmsd) in purple (negative)
and green (positive)

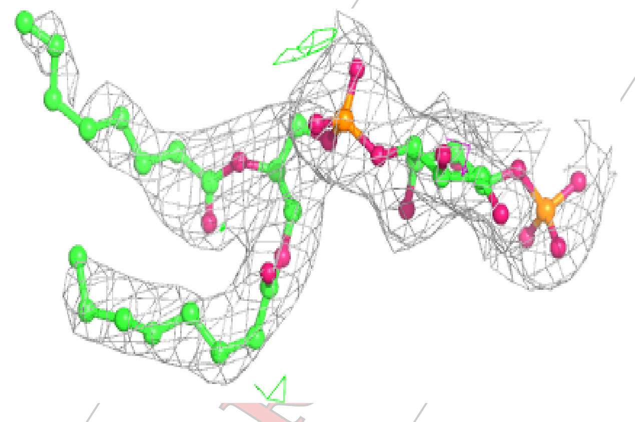

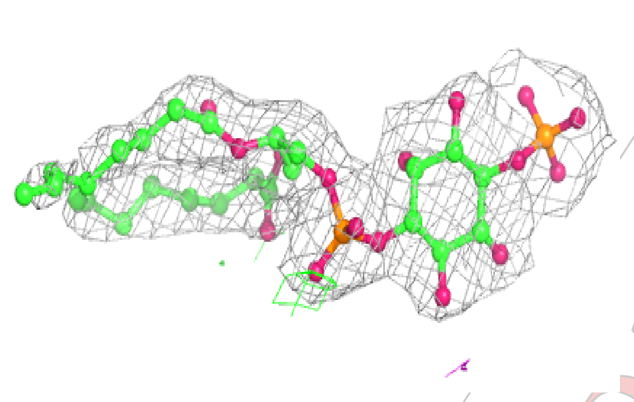

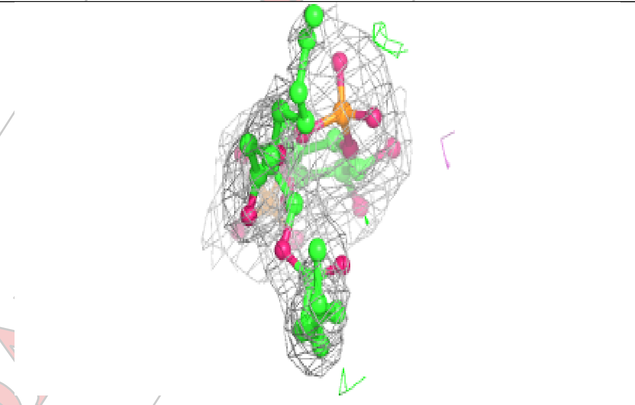

6.5 Other polymers (i)

There are no such residues in this entry.

PRELIMINARY VALIDATION
