# Supplementary material for: Structure of human ORP3 ORD reveals conservation of a key function and ligand specificity in OSBP-related proteins
Source: PLoS One. 2021 Apr 15;16(4):e0248781. doi: 10.1371/journal.pone.0248781 (PMC8049286; doi:10.1371/journal.pone.0248781)
Supplement: S2 File — (PDF) [file pone.0248781.s002.pdf]

Preliminary Full wwPDB X-ray Structure Validation Report (i)

Nov 4, 2020 – 01:00 PM JST

Deposition ID : D_1300019247

This is a Preliminary Full wwPDB X-ray Structure Validation Report.

This report is produced by the wwPDB Deposition System during initial deposition but before annotation of the structure.

We welcome your comments at *validation@mail.wwpdb.org*
A user guide is available at
https://www.wwpdb.org/validation/2017/XrayValidationReportHelp
with specific help available everywhere you see the (i) symbol.

The following versions of software and data (see references (i)) were used in the production of this report:

MolProbity	:	4.02b-467
Xtriage (Phenix)	:	1.13
EDS	:	2.14.6
Percentile statistics	:	20191225.v01 (using entries in the PDB archive December 25th 2019)
Refmac	:	5.8.0158
CCP4	:	7.0.044 (Gargrove)
Ideal geometry (proteins)	:	Engh & Huber (2001)
Ideal geometry (DNA, RNA)	:	Parkinson et al. (1996)
Validation Pipeline (wwPDB-VP)	:	2.14.6

PRELIMINARY VALIDATION REPORT

1 Overall quality at a glance (i)

The following experimental techniques were used to determine the structure:
X-RAY DIFFRACTION

The reported resolution of this entry is 2.70 Å.

Percentile scores (ranging between 0-100) for global validation metrics of the entry are shown in the following graphic. The table shows the number of entries on which the scores are based.

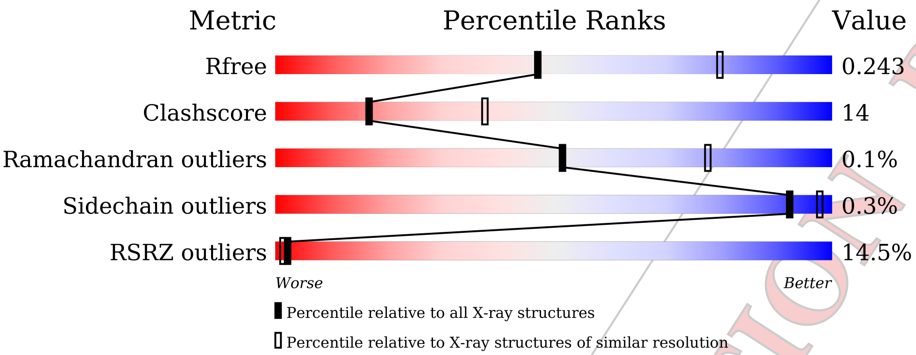

Metric	Whole archive (#Entries)	Similar resolution (#Entries, resolution range(Å))
R_{free}	130704	2808 (2.70-2.70)
Clashscore	141614	3122 (2.70-2.70)
Ramachandran outliers	138981	3069 (2.70-2.70)
Sidechain outliers	138945	3069 (2.70-2.70)
RSRZ outliers	127900	2737 (2.70-2.70)

The table below summarises the geometric issues observed across the polymeric chains and their fit to the electron density. The red, orange, yellow and green segments on the lower bar indicate the fraction of residues that contain outliers for >=3, 2, 1 and 0 types of geometric quality criteria respectively. A grey segment represents the fraction of residues that are not modelled. The numeric value for each fraction is indicated below the corresponding segment, with a dot representing fractions <=5% The upper red bar (where present) indicates the fraction of residues that have poor fit to the electron density. The numeric value is given above the bar.

Mol	Chain	Length	Quality of chain
1	A	375	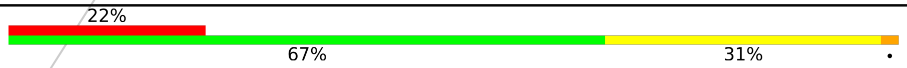
2	B	376	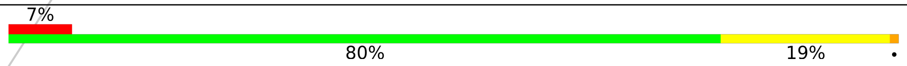

PRELIMINARY VALIDATION REPORT

2 Entry composition (i)

There are 3 unique types of molecules in this entry. The entry contains 6210 atoms, of which 0 are hydrogens and 0 are deuteriums.

In the tables below, the ZeroOcc column contains the number of atoms modelled with zero occupancy, the AltConf column contains the number of residues with at least one atom in alternate conformation and the Trace column contains the number of residues modelled with at most 2 atoms.

- Molecule 1 is a protein.

Mol	Chain	Residues	Atoms					ZeroOcc	AltConf	Trace
1	A	375	Total<br>3072	C<br>1955	N<br>540	O<br>564	S<br>13	0	0	0

- Molecule 2 is a protein.

Mol	Chain	Residues	Atoms					ZeroOcc	AltConf	Trace
2	B	376	Total<br>3083	C<br>1961	N<br>544	O<br>565	S<br>13	0	0	0

- Molecule 3 is water.

Mol	Chain	Residues	Atoms		ZeroOcc	AltConf
3	S	55	Total<br>55	O<br>55	0	0

PRELIMINARY VALIDATION REPORT

3 Residue-property plots (i)

These plots are drawn for all protein, RNA, DNA and oligosaccharide chains in the entry. The first graphic for a chain summarises the proportions of the various outlier classes displayed in the second graphic. The second graphic shows the sequence view annotated by issues in geometry and electron density. Residues are color-coded according to the number of geometric quality criteria for which they contain at least one outlier: green = 0, yellow = 1, orange = 2 and red = 3 or more. A red dot above a residue indicates a poor fit to the electron density (RSRZ > 2). Stretches of 2 or more consecutive residues without any outlier are shown as a green connector. Residues present in the sample, but not in the model, are shown in grey.

- Molecule 1:

Chain A:

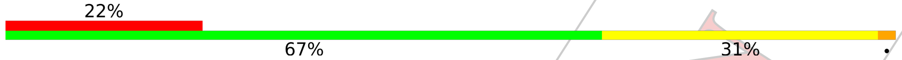

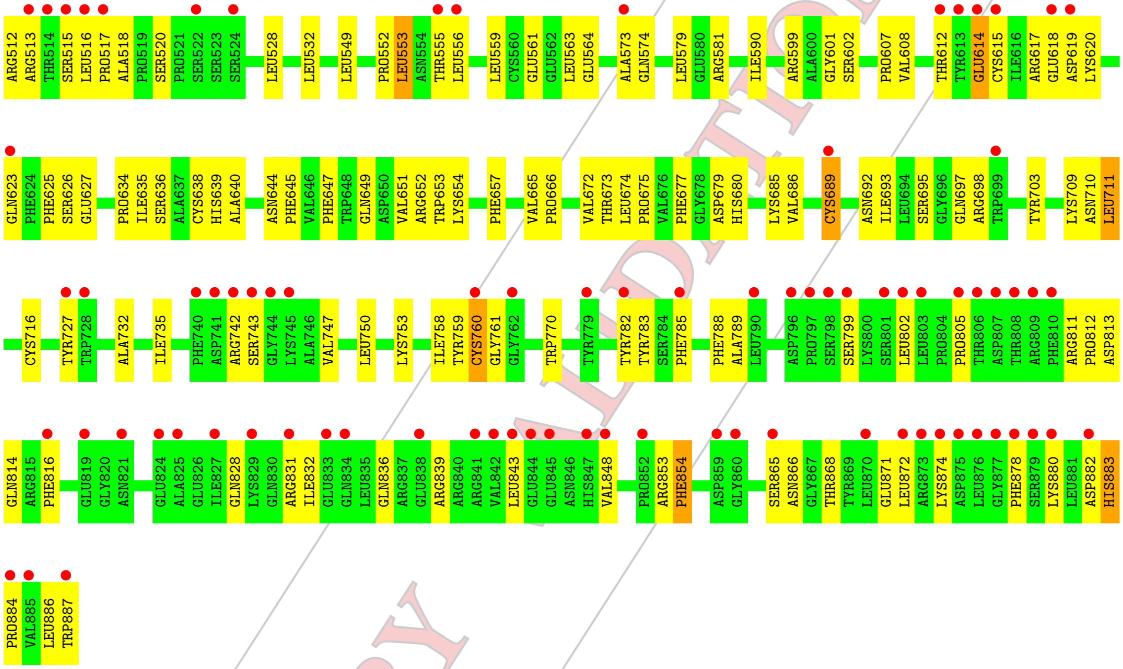

- Molecule 2:

Chain B:

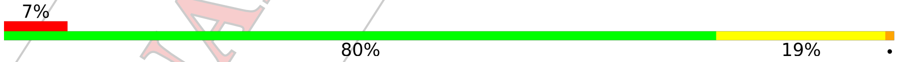

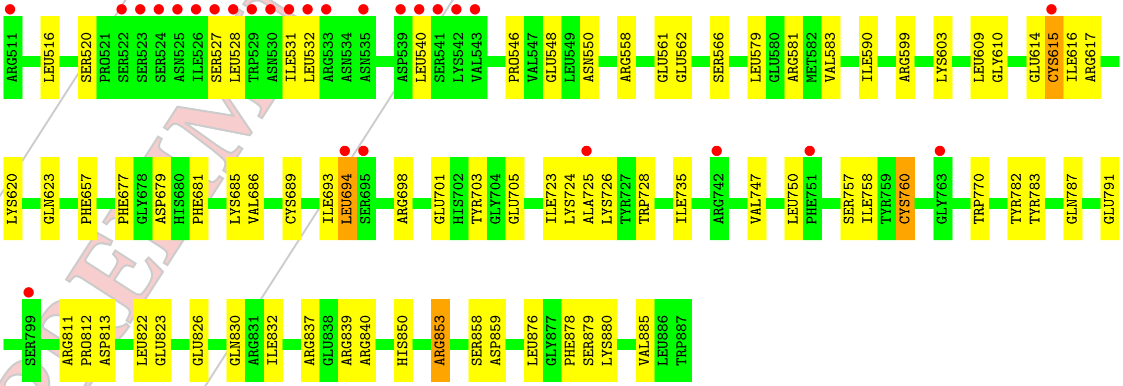

PRELIMINARY VALIDATION REPORT

4 Data and refinement statistics (i)

Property	Value	Source
Space group	P 31 2 1	Depositor
Cell constants<br>a, b, c, α, β, γ	95.91Å 95.91Å 188.90Å<br>90.00° 90.00° 120.00°	Depositor
Resolution (Å)	29.44 – 2.70<br>29.44 – 2.70	Depositor<br>EDS
% Data completeness<br>(in resolution range)	98.9 (29.44-2.70)<br>98.9 (29.44-2.70)	Depositor<br>EDS
R_{merge}	(Not available)	Depositor
R_{sym}	(Not available)	Depositor
$< I/\sigma(I) >$ [1]	5.93 (at 2.72Å)	Xtriage
Refinement program	phenix.refine 1.18.2_3874, PHENIX 1.18.2_3874	Depositor
R, R_{free}	0.233 , 0.285<br>0.235 , 0.243	Depositor<br>DCC
R_{free} test set	1417 reflections (5.06%)	wwPDB-VP
Wilson B-factor (Å^2)	57.6	Xtriage
Anisotropy	0.034	Xtriage
Bulk solvent k_{sol}(e/Å^3), B_{sol}(Å^2)	0.34 , 60.9	EDS
L-test for twinning[2]	$< \|L\| > = 0.49$, $< L^2 > = 0.32$	Xtriage
Estimated twinning fraction	0.028 for -h,-k,l	Xtriage
F_o,F_c correlation	0.92	EDS
Total number of atoms	6210	wwPDB-VP
Average B, all atoms (Å^2)	70.0	wwPDB-VP

Xtriage's analysis on translational NCS is as follows: *The largest off-origin peak in the Patterson function is 3.83% of the height of the origin peak. No significant pseudotranslation is detected.*

[1] Intensities estimated from amplitudes.

[2] Theoretical values of $< |L| >$, $< L^2 >$ for acentric reflections are 0.5, 0.333 respectively for untwinned datasets, and 0.375, 0.2 for perfectly twinned datasets.

PRELIMINARY VALIDATION REPORT

5 Model quality (i)

5.1 Standard geometry (i)

The Z score for a bond length (or angle) is the number of standard deviations the observed value is removed from the expected value. A bond length (or angle) with $|Z| > 5$ is considered an outlier worth inspection. RMSZ is the root-mean-square of all Z scores of the bond lengths (or angles).

Mol	Chain	Bond lengths		Bond angles	
		RMSZ	#$\|Z\|$ >5	RMSZ	#$\|Z\|$ >5
1	A	0.57	5/3162 (0.2%)	0.74	6/4284 (0.1%)
2	B	0.69	2/3173 (0.1%)	0.74	2/4298 (0.0%)
All	All	0.63	7/6335 (0.1%)	0.74	8/8582 (0.1%)

Chiral center outliers are detected by calculating the chiral volume of a chiral center and verifying if the center is modelled as a planar moiety or with the opposite hand.A planarity outlier is detected by checking planarity of atoms in a peptide group, atoms in a mainchain group or atoms of a sidechain that are expected to be planar.

Mol	Chain	#Chirality outliers	#Planarity outliers
1	A	0	3
2	B	0	1
All	All	0	4

All (7) bond length outliers are listed below:

Mol	Chain	Res	Type	Atoms	Z	Observed(Å)	Ideal(Å)
2	B	615	CYS	CB-SG	16.56	2.10	1.82
2	B	760	CYS	CB-SG	11.95	2.02	1.82
1	A	760	CYS	CB-SG	10.36	1.99	1.82
1	A	689	CYS	CB-SG	10.03	1.99	1.82
1	A	614	GLU	CD-OE1	7.58	1.33	1.25
1	A	618	GLU	CD-OE1	-5.59	1.19	1.25
1	A	614	GLU	CD-OE2	5.23	1.31	1.25

All (8) bond angle outliers are listed below:

Mol	Chain	Res	Type	Atoms	Z	Observed(^{o})	Ideal(^{o})
1	A	689	CYS	CA-CB-SG	7.77	127.99	114.00
1	A	614	GLU	CG-CD-OE1	-7.62	103.06	118.30
2	B	760	CYS	CA-CB-SG	7.61	127.70	114.00
2	B	694	LEU	CA-CB-CG	5.80	128.63	115.30
1	A	711	LEU	CB-CG-CD2	-5.74	101.24	111.00

Continued on next page...

PRELIMINARY VALIDATION REPORT

Continued from previous page...

Mol	Chain	Res	Type	Atoms	Z	Observed(^{o})	Ideal(^{o})
1	A	634	PRO	N-CD-CG	5.50	111.46	103.20
1	A	553	LEU	CA-CB-CG	5.36	127.62	115.30
1	A	614	GLU	N-CA-C	-5.01	97.48	111.00

There are no chirality outliers.

All (4) planarity outliers are listed below:

Mol	Chain	Res	Type	Group
1	A	601	GLY	Peptide
1	A	614	GLU	Sidechain
1	A	883	HIS	Peptide
2	B	858	SER	Peptide

5.2 Too-close contacts (i)

In the following table, the Non-H and H(model) columns list the number of non-hydrogen atoms and hydrogen atoms in the chain respectively. The H(added) column lists the number of hydrogen atoms added and optimized by MolProbity. The Clashes column lists the number of clashes within the asymmetric unit, whereas Symm-Clashes lists symmetry related clashes.

Mol	Chain	Non-H	H(model)	H(added)	Clashes	Symm-Clashes
1	A	3072	0	2959	100	0
2	B	3083	0	2972	70	0
3	S	55	0	0	28	0
All	All	6210	0	5931	170	0

The all-atom clashscore is defined as the number of clashes found per 1000 atoms (including hydrogen atoms). The all-atom clashscore for this structure is 14.

All (170) close contacts within the same asymmetric unit are listed below, sorted by their clash magnitude.

Atom-1	Atom-2	Interatomic distance (Å)	Clash overlap (Å)
2:B:760:CYS:CB	2:B:760:CYS:SG	2.02	1.47
2:B:615:CYS:SG	2:B:615:CYS:CB	2.10	1.39
2:B:725:ALA:HB1	3:S:51:HOH:O	1.40	1.21
2:B:558:ARG:HG2	3:S:53:HOH:O	1.52	1.09
2:B:726:LYS:N	3:S:51:HOH:O	1.84	1.08
2:B:760:CYS:SG	3:S:11:HOH:O	2.24	0.95
2:B:725:ALA:CB	3:S:51:HOH:O	2.03	0.90

Continued on next page...

PRELIMINARY VALIDATION REPORT

Continued from previous page...

Atom-1	Atom-2	Interatomic distance (Å)	Clash overlap (Å)
2:B:726:LYS:O	3:S:51:HOH:O	1.92	0.87
2:B:540:LEU:HD11	2:B:657:PHE:HB2	1.58	0.86
2:B:561:GLU:OE1	3:S:53:HOH:O	1.95	0.84
1:A:513:ARG:HE	1:A:515:SER:HB2	1.40	0.84
2:B:527:SER:HB3	2:B:546:PRO:HG2	1.60	0.84
2:B:735:ILE:HD11	2:B:758:ILE:HD12	1.59	0.84
1:A:832:ILE:O	1:A:836:GLN:NE2	2.11	0.84
1:A:602:SER:O	3:S:48:HOH:O	1.95	0.83
1:A:617:ARG:HH11	1:A:619:ASP:HB3	1.46	0.79
1:A:581:ARG:NH2	1:A:679:ASP:OD2	2.17	0.78
2:B:876:LEU:O	3:S:39:HOH:O	2.03	0.77
1:A:716:CYS:SG	3:S:32:HOH:O	2.42	0.77
2:B:617:ARG:NH2	2:B:791:GLU:OE1	2.19	0.74
2:B:879:SER:HB2	3:S:39:HOH:O	1.88	0.74
1:A:615:CYS:SG	3:S:10:HOH:O	2.44	0.74
2:B:876:LEU:C	3:S:38:HOH:O	2.27	0.73
2:B:724:LYS:O	3:S:49:HOH:O	2.06	0.72
1:A:602:SER:CB	3:S:48:HOH:O	2.38	0.71
1:A:602:SER:HB2	3:S:48:HOH:O	1.89	0.71
1:A:617:ARG:HG2	1:A:620:LYS:H	1.56	0.71
2:B:614:GLU:OE2	3:S:9:HOH:O	2.10	0.70
2:B:686:VAL:HG13	2:B:703:TYR:O	1.92	0.69
2:B:812:PRO:HD2	2:B:832:ILE:HD11	1.73	0.69
1:A:686:VAL:HG22	1:A:703:TYR:O	1.93	0.69
1:A:617:ARG:HD3	1:A:620:LYS:HG3	1.76	0.68
2:B:703:TYR:HB3	2:B:724:LYS:HB2	1.76	0.67
1:A:853:ARG:NH1	1:A:883:HIS:HB2	2.11	0.66
1:A:602:SER:CA	3:S:48:HOH:O	2.43	0.66
2:B:823:GLU:OE1	3:S:30:HOH:O	2.14	0.66
2:B:617:ARG:HH11	2:B:620:LYS:HG3	1.60	0.66
1:A:653:TRP:CZ3	1:A:666:PRO:HG3	2.31	0.66
1:A:871:GLU:N	1:A:871:GLU:OE1	2.30	0.65
1:A:692:ASN:HB3	1:A:695:SER:HB2	1.79	0.65
1:A:828:GLN:O	1:A:832:ILE:HD12	1.98	0.64
2:B:878:PHE:N	3:S:38:HOH:O	2.32	0.62
2:B:520:SER:HB2	2:B:783:TYR:H	1.64	0.62
1:A:602:SER:C	3:S:48:HOH:O	2.36	0.62
1:A:854:PHE:HA	1:A:866:ASN:HD21	1.65	0.61
1:A:635:ILE:HG12	1:A:652:ARG:HB3	1.82	0.60
1:A:785:PHE:HB3	1:A:789:ALA:HB3	1.83	0.60
2:B:758:ILE:HG12	2:B:770:TRP:HE3	1.67	0.60

Continued on next page...

PRELIMINARY VALIDATION REPORT

Continued from previous page...

Atom-1	Atom-2	Interatomic distance (Å)	Clash overlap (Å)
1:A:758:ILE:HG12	1:A:770:TRP:HE3	1.66	0.59
2:B:725:ALA:HA	3:S:49:HOH:O	2.03	0.58
2:B:562:GLU:OE1	2:B:685:LYS:NZ	2.35	0.58
2:B:837:ARG:HG2	2:B:840:ARG:HH22	1.68	0.58
1:A:697:GLN:HG3	1:A:727:TYR:CE2	2.40	0.57
1:A:625:PHE:CZ	1:A:639:HIS:HB3	2.39	0.56
1:A:513:ARG:HG3	1:A:515:SER:H	1.68	0.56
1:A:758:ILE:HG12	1:A:770:TRP:CE3	2.40	0.56
2:B:548:GLU:HG2	2:B:822:LEU:HD12	1.87	0.55
2:B:528:LEU:HD12	2:B:532:LEU:HD13	1.88	0.55
1:A:627:GLU:OE2	1:A:853:ARG:HB2	2.06	0.55
2:B:590:ILE:HD13	2:B:735:ILE:HD12	1.87	0.55
1:A:673:THR:HG23	1:A:680:HIS:HD2	1.70	0.55
1:A:617:ARG:HH11	1:A:619:ASP:CB	2.19	0.55
1:A:680:HIS:ND1	1:A:711:LEU:HD12	2.21	0.55
1:A:843:LEU:HB2	1:A:848:VAL:HB	1.88	0.55
2:B:616:ILE:HG23	2:B:623:GLN:HG2	1.89	0.55
1:A:528:LEU:HG	1:A:549:LEU:HD13	1.88	0.54
1:A:513:ARG:NE	1:A:515:SER:HB2	2.19	0.54
1:A:561:GLU:OE2	1:A:599:ARG:NH1	2.40	0.54
1:A:512:ARG:NH2	1:A:617:ARG:HH12	2.06	0.54
2:B:583:VAL:HG13	2:B:750:LEU:HD21	1.89	0.54
2:B:579:LEU:HD22	2:B:747:VAL:HG21	1.90	0.53
1:A:579:LEU:HD13	1:A:747:VAL:HG11	1.91	0.52
1:A:805:PRO:HG3	1:A:882:ASP:OD2	2.09	0.52
1:A:579:LEU:CD2	1:A:716:CYS:HB3	2.40	0.51
1:A:812:PRO:HB2	1:A:828:GLN:HG3	1.92	0.51
2:B:735:ILE:HD11	2:B:758:ILE:CD1	2.36	0.51
2:B:693:ILE:O	2:B:694:LEU:HG	2.10	0.51
2:B:561:GLU:OE2	2:B:599:ARG:NH1	2.41	0.51
2:B:723:ILE:CG2	3:S:49:HOH:O	2.57	0.51
2:B:757:SER:O	2:B:758:ILE:HD13	2.12	0.50
1:A:573:ALA:HB2	1:A:674:LEU:HB3	1.93	0.50
1:A:612:THR:HG22	1:A:627:GLU:HG3	1.92	0.50
2:B:701:GLU:OE1	2:B:724:LYS:NZ	2.43	0.50
1:A:552:PRO:O	1:A:553:LEU:HD22	2.12	0.50
1:A:556:LEU:HD23	1:A:636:SER:HB3	1.93	0.50
1:A:799:SER:HA	1:A:802:LEU:HD12	1.92	0.50
1:A:883:HIS:HB3	1:A:884:PRO:HD2	1.92	0.49
2:B:531:ILE:HG22	2:B:532:LEU:HD12	1.95	0.49
2:B:689:CYS:HB2	2:B:703:TYR:OH	2.11	0.49

Continued on next page...

PRELIMINARY VALIDATION REPORT

Continued from previous page...

Atom-1	Atom-2	Interatomic distance (Å)	Clash overlap (Å)
1:A:812:PRO:O	1:A:816:PHE:HD1	1.94	0.49
1:A:831:ARG:HH22	1:A:887:TRP:HA	1.77	0.49
2:B:590:ILE:HD13	2:B:735:ILE:CD1	2.42	0.49
2:B:876:LEU:O	3:S:38:HOH:O	2.19	0.49
1:A:653:TRP:HZ3	1:A:666:PRO:HG3	1.75	0.48
1:A:871:GLU:H	1:A:871:GLU:CD	2.09	0.48
1:A:882:ASP:N	1:A:882:ASP:OD1	2.47	0.48
1:A:553:LEU:HD23	1:A:785:PHE:CZ	2.48	0.48
2:B:579:LEU:CD2	2:B:747:VAL:HG21	2.44	0.48
2:B:811:ARG:HG2	2:B:813:ASP:OD1	2.13	0.48
2:B:516:LEU:HB2	2:B:782:TYR:CD1	2.49	0.48
1:A:532:LEU:HB3	1:A:657:PHE:CD2	2.49	0.48
1:A:608:VAL:HG11	1:A:886:LEU:HD12	1.95	0.48
1:A:871:GLU:HA	1:A:874:LYS:HB2	1.96	0.47
2:B:528:LEU:HD22	2:B:698:ARG:NH1	2.29	0.47
2:B:837:ARG:HG2	2:B:840:ARG:NH2	2.29	0.47
1:A:608:VAL:HG23	1:A:811:ARG:NH1	2.29	0.47
1:A:673:THR:HA	1:A:679:ASP:O	2.14	0.47
2:B:705:GLU:N	3:S:1:HOH:O	2.17	0.47
2:B:609:LEU:HD11	2:B:850:HIS:CD2	2.50	0.47
2:B:826:GLU:O	2:B:830:GLN:HG3	2.14	0.47
1:A:559:LEU:HD11	1:A:651:VAL:HG22	1.96	0.47
1:A:673:THR:HG22	1:A:680:HIS:HA	1.96	0.47
1:A:878:PHE:CE2	1:A:880:LYS:HD2	2.50	0.47
2:B:566:SER:OG	2:B:620:LYS:HE3	2.15	0.47
1:A:697:GLN:O	1:A:697:GLN:HG2	2.15	0.47
2:B:839:ARG:NH2	2:B:885:VAL:H	2.14	0.46
1:A:868:THR:O	1:A:872:LEU:HB2	2.15	0.46
1:A:654:LYS:HB3	1:A:665:VAL:HB	1.96	0.46
2:B:726:LYS:HE2	2:B:728:TRP:HE1	1.80	0.46
1:A:697:GLN:HG3	1:A:727:TYR:CZ	2.50	0.46
2:B:528:LEU:HD21	2:B:694:LEU:H	1.79	0.46
1:A:520:SER:HB2	1:A:783:TYR:CD1	2.51	0.46
2:B:726:LYS:HB3	2:B:728:TRP:NE1	2.31	0.46
2:B:703:TYR:CB	2:B:724:LYS:HB2	2.46	0.45
1:A:709:LYS:HE3	1:A:709:LYS:HB2	1.55	0.45
1:A:556:LEU:CD2	1:A:636:SER:HB3	2.46	0.45
1:A:647:PHE:HD1	1:A:672:VAL:HG22	1.80	0.45
1:A:854:PHE:HA	1:A:866:ASN:ND2	2.31	0.45
1:A:813:ASP:OD1	1:A:814:GLN:N	2.50	0.45
1:A:555:THR:HG23	1:A:607:PRO:HD3	1.99	0.45

Continued on next page...

PRELIMINARY VALIDATION REPORT

Continued from previous page...

Atom-1	Atom-2	Interatomic distance (Å)	Clash overlap (Å)
1:A:608:VAL:HG13	1:A:836:GLN:HG2	1.99	0.45
2:B:878:PHE:C	2:B:880:LYS:H	2.20	0.45
1:A:617:ARG:HD3	1:A:620:LYS:CG	2.46	0.44
1:A:645:PHE:HA	1:A:673:THR:O	2.17	0.44
1:A:760:CYS:SG	1:A:761:GLY:N	2.91	0.44
1:A:625:PHE:HZ	1:A:865:SER:HG	1.64	0.44
2:B:610:GLY:O	2:B:853:ARG:NH1	2.50	0.44
2:B:724:LYS:C	3:S:49:HOH:O	2.51	0.44
1:A:516:LEU:O	1:A:518:ALA:N	2.51	0.44
1:A:623:GLN:O	1:A:640:ALA:HA	2.18	0.44
1:A:573:ALA:HB2	1:A:674:LEU:HD22	2.01	0.43
1:A:563:LEU:HD21	1:A:647:PHE:CD2	2.53	0.43
1:A:574:GLN:NE2	1:A:644:ASN:HD22	2.16	0.43
1:A:693:ILE:HA	1:A:698:ARG:HG2	2.00	0.43
1:A:590:ILE:HD13	1:A:735:ILE:HD13	2.01	0.42
1:A:742:ARG:HG3	1:A:743:SER:N	2.34	0.42
2:B:878:PHE:O	2:B:880:LYS:N	2.49	0.42
1:A:638:CYS:SG	1:A:649:GLN:HG2	2.60	0.42
2:B:758:ILE:HG12	2:B:770:TRP:CE3	2.50	0.42
1:A:839:ARG:O	1:A:843:LEU:HG	2.20	0.41
1:A:581:ARG:NH2	1:A:679:ASP:CG	2.72	0.41
1:A:556:LEU:HB3	1:A:626:SER:OG	2.20	0.41
1:A:673:THR:CG2	1:A:680:HIS:CD2	3.03	0.41
1:A:515:SER:HA	1:A:782:TYR:HE2	1.84	0.41
1:A:680:HIS:O	1:A:710:ASN:HA	2.21	0.41
1:A:564:GLU:HA	1:A:788:PHE:CE2	2.56	0.41
1:A:750:LEU:HA	1:A:759:TYR:O	2.21	0.41
2:B:558:ARG:CG	3:S:53:HOH:O	2.35	0.41
2:B:679:ASP:HB3	2:B:681:PHE:CE2	2.56	0.41
1:A:813:ASP:HB3	1:A:828:GLN:HB2	2.02	0.41
1:A:649:GLN:OE1	1:A:685:LYS:HE3	2.21	0.41
2:B:581:ARG:HD3	2:B:677:PHE:CE2	2.56	0.41
1:A:673:THR:HG23	1:A:680:HIS:CD2	2.53	0.40
2:B:725:ALA:CA	3:S:49:HOH:O	2.65	0.40
1:A:675:PRO:O	1:A:677:PHE:N	2.54	0.40
1:A:732:ALA:O	1:A:753:LYS:HE3	2.21	0.40
1:A:516:LEU:HG	1:A:517:PRO:HD2	2.04	0.40
2:B:550:ASN:HA	2:B:603:LYS:O	2.21	0.40
1:A:689:CYS:HB2	1:A:703:TYR:CE1	2.57	0.40
2:B:787:GLN:O	2:B:791:GLU:HG3	2.22	0.40

There are no symmetry-related clashes.

PRELIMINARY VALIDATION REPORT

5.3 Torsion angles (i)

5.3.1 Protein backbone (i)

In the following table, the Percentiles column shows the percent Ramachandran outliers of the chain as a percentile score with respect to all X-ray entries followed by that with respect to entries of similar resolution.

The Analysed column shows the number of residues for which the backbone conformation was analysed, and the total number of residues.

Mol	Chain	Analysed	Favoured	Allowed	Outliers	Percentiles	
1	A	373/375 (100%)	342 (92%)	31 (8%)	0	100	100
2	B	374/376 (100%)	351 (94%)	22 (6%)	1 (0%)	41	66
All	All	747/751 (100%)	693 (93%)	53 (7%)	1 (0%)	51	78

All (1) Ramachandran outliers are listed below:

Mol	Chain	Res	Type
2	B	859	ASP

5.3.2 Protein sidechains (i)

In the following table, the Percentiles column shows the percent sidechain outliers of the chain as a percentile score with respect to all X-ray entries followed by that with respect to entries of similar resolution.

The Analysed column shows the number of residues for which the sidechain conformation was analysed, and the total number of residues.

Mol	Chain	Analysed	Rotameric	Outliers	Percentiles	
1	A	336/336 (100%)	335 (100%)	1 (0%)	92	98
2	B	337/337 (100%)	336 (100%)	1 (0%)	92	98
All	All	673/673 (100%)	671 (100%)	2 (0%)	92	98

All (2) residues with a non-rotameric sidechain are listed below:

Mol	Chain	Res	Type
1	A	854	PHE
2	B	853	ARG

Some sidechains can be flipped to improve hydrogen bonding and reduce clashes. All (4) such sidechains are listed below:

PRELIMINARY VALIDATION REPORT

Mol	Chain	Res	Type
1	A	644	ASN
1	A	836	GLN
1	A	851	GLN
2	B	850	HIS

5.3.3 RNA (i)

There are no RNA molecules in this entry.

5.4 Non-standard residues in protein, DNA, RNA chains (i)

There are no non-standard protein/DNA/RNA residues in this entry.

5.5 Carbohydrates (i)

There are no monosaccharides in this entry.

5.6 Ligand geometry (i)

There are no ligands in this entry.

5.7 Other polymers (i)

There are no such residues in this entry.

5.8 Polymer linkage issues (i)

There are no chain breaks in this entry.

PRELIMINARY VALIDATION REPORT

6 Fit of model and data (i)

6.1 Protein, DNA and RNA chains (i)

In the following table, the column labelled '#RSRZ> 2' contains the number (and percentage) of RSRZ outliers, followed by percent RSRZ outliers for the chain as percentile scores relative to all X-ray entries and entries of similar resolution. The OWAB column contains the minimum, median, 95th percentile and maximum values of the occupancy-weighted average B-factor per residue. The column labelled 'Q< 0.9' lists the number of (and percentage) of residues with an average occupancy less than 0.9.

Mol	Chain	Analysed	<RSRZ>	#RSRZ>2			OWAB($Å^2$)	Q<0.9
1	A	375/375 (100%)	1.09	82 (21%)	0	0	40, 88, 131, 142	0
2	B	376/376 (100%)	0.19	27 (7%)	15	13	27, 48, 106, 131	0
All	All	751/751 (100%)	0.64	109 (14%)	2	1	27, 64, 124, 142	0

All (109) RSRZ outliers are listed below:

Mol	Chain	Res	Type	RSRZ
2	B	541	SER	7.7
2	B	527	SER	6.7
1	A	885	VAL	6.5
1	A	847	HIS	5.9
1	A	728	TRP	5.7
1	A	522	SER	5.6
1	A	802	LEU	5.4
1	A	833	GLU	5.3
1	A	860	GLY	5.2
1	A	876	LEU	5.1
2	B	529	TRP	4.9
2	B	524	SER	4.9
1	A	870	LEU	4.8
2	B	694	LEU	4.7
2	B	763	GLY	4.6
2	B	542	LYS	4.6
1	A	843	LEU	4.6
2	B	530	ASN	4.5
1	A	873	ARG	4.4
1	A	848	VAL	4.4
2	B	531	ILE	4.4
1	A	887	TRP	4.3
1	A	612	THR	4.2
1	A	875	ASP	4.1

Continued on next page...

PRELIMINARY VALIDATION REPORT

Continued from previous page...

Mol	Chain	Res	Type	RSRZ
1	A	514	THR	4.0
1	A	613	TYR	3.9
2	B	526	ILE	3.9
1	A	841	ARG	3.8
1	A	801	SER	3.6
1	A	816	PHE	3.6
2	B	539	ASP	3.6
2	B	522	SER	3.6
2	B	525	ASN	3.6
1	A	727	TYR	3.4
1	A	745	LYS	3.4
1	A	513	ARG	3.3
2	B	742	ARG	3.3
1	A	882	ASP	3.3
2	B	532	LEU	3.3
1	A	824	GLU	3.2
1	A	805	PRO	3.2
1	A	845	GLU	3.2
1	A	878	PHE	3.2
1	A	819	GLU	3.2
2	B	523	SER	3.2
1	A	842	VAL	3.1
1	A	742	ARG	3.1
1	A	515	SER	3.1
2	B	725	ALA	3.1
1	A	872	LEU	3.1
1	A	877	GLY	3.0
1	A	615	CYS	3.0
1	A	556	LEU	3.0
1	A	517	PRO	3.0
1	A	782	TYR	3.0
1	A	880	LYS	2.9
1	A	859	ASP	2.8
1	A	825	ALA	2.8
1	A	744	GLY	2.8
1	A	852	PRO	2.7
1	A	762	GLY	2.7
1	A	740	PHE	2.7
1	A	808	THR	2.7
1	A	799	SER	2.7
1	A	743	SER	2.6
1	A	865	SER	2.6

Continued on next page...

PRELIMINARY VALIDATION REPORT

Continued from previous page...

Mol	Chain	Res	Type	RSRZ
1	A	821	ASN	2.6
1	A	555	THR	2.6
1	A	516	LEU	2.6
2	B	615	CYS	2.5
1	A	741	ASP	2.5
1	A	807	ASP	2.5
2	B	695	SER	2.5
2	B	533	ARG	2.5
2	B	799	SER	2.5
1	A	803	LEU	2.5
2	B	535	ASN	2.5
2	B	511	ARG	2.4
1	A	874	LYS	2.4
1	A	827	ILE	2.4
1	A	806	THR	2.4
2	B	528	LEU	2.4
1	A	798	SER	2.3
1	A	834	GLN	2.3
1	A	573	ALA	2.3
2	B	751	PHE	2.3
1	A	699	TRP	2.3
1	A	797	PRO	2.3
1	A	844	GLU	2.3
1	A	760	CYS	2.3
1	A	796	ASP	2.3
1	A	831	ARG	2.3
1	A	810	PHE	2.3
1	A	618	GLU	2.3
1	A	524	SER	2.2
1	A	623	GLN	2.2
1	A	619	ASP	2.2
1	A	614	GLU	2.1
1	A	884	PRO	2.1
1	A	879	SER	2.1
1	A	809	ARG	2.1
1	A	779	TYR	2.1
1	A	838	GLU	2.1
2	B	543	VAL	2.1
1	A	785	PHE	2.1
2	B	540	LEU	2.0
1	A	790	LEU	2.0
1	A	689	CYS	2.0

Continued on next page...

PRELIMINARY VALIDATION REPORT

Continued from previous page...

Mol	Chain	Res	Type	RSRZ
1	A	829	LYS	2.0

6.2 Non-standard residues in protein, DNA, RNA chains (i)

There are no non-standard protein/DNA/RNA residues in this entry.

6.3 Carbohydrates (i)

There are no monosaccharides in this entry.

6.4 Ligands (i)

There are no ligands in this entry.

6.5 Other polymers (i)

There are no such residues in this entry.

PRELIMINARY VALIDATION REPORT
