# Supplementary material for: Structure of human ORP3 ORD reveals conservation of a key function and ligand specificity in OSBP-related proteins
Source: PLoS One. 2021 Apr 15;16(4):e0248781. doi: 10.1371/journal.pone.0248781 (PMC8049286; doi:10.1371/journal.pone.0248781)
Supplement: Attachment — Submitted filename: Response to reviewers.docx [file pone.0248781.s003.docx]

**Response to reviewer’s comments**

We appreciate the editor and reviewer’s constructive and insightful comments. We have addresses the criticisms in the revised manuscript as suggested.

**Editor:**

Please address all the suggested changes as described by each reviewer below, and please provide in a separate document an explanation of revisions made. For Fig.4C, please include wild-type controls for all plasmid transformations. It is presumed that all the constructs will either rescue the osh∆ osh4-1 growth defect or not. Some of these constructs could actually cause dominant growth defects, which would completely change the interpretation of these results.

>> The CBY926 cells were transformed with the pRS416MET plasmids containing GFP-ORP constructs. The identical constructs were used for yeast complementation test and cell imaging. The difference between two experiments was growth temperatures. The complementation test was performed at 37℃ and the yeast imaging was at 30℃. The yeast cells for imaging were grown at a permissive temperature to allow growth of the yeast cells harboring the non-complementing ORP3 constructs. All constructs used for the experiments grew well at 30℃. To prepare stock cells for the dilution series, the transformed cells were grown to the optical density around 1 and the slight difference of cell densities between the constructs were normalized for the preparation of the dilution series. Then, the yeast cells were spotted on the agar plates and grown at 37℃ (restrictive temperature). Since all constructs grew well at permissive temperature (30℃), which is shown in the yeast imaging (Fig 4B). If any of the constructs had dominant growth defects, we could not have obtained the yeast transformants for the experiments. This indicates that the human ORP3 constructs did not cause any dominant growth defects. Therefore, I do not think that it is necessary to show the growth of all constructs at 30℃. I think that empty plasmid and full length ORP3 (FL) serve as good internal controls for this purpose. We added the detailed description in the revised manuscript to avoid confusion.

(3) Fig.4B, representative cell images are provided but no quantification is provided. How many cells were counted? What percentage of these cells exhibited punctate or cytoplasmic localization? Are the differences statistically significant?

>> Generally, we inspected more than 100 yeast cells for each constructs and showed representative images for the cells. They showed quite consistent localizations for the cell population of the same constructs. We could not perform quantitative analysis of cell imaging and statistics due to our technical limitations. I believe that the main conclusion of protein-targeting and functional complementation in yeast cells is clear without quantification of cell imaging.

**Reviewer #1:**

This work reports the structure of ORP3-ORD. This is an important contribution to the study of ORPs. There are only some minor concerns.

1. The structures of ORP3-ORD, alone and in complex with PI4P, have been reported (BBRC 529 (2020) 1005-1010). Please compare your structures with the reported ones, and show the structural differences.

>> We have included a figure (Figure 2D) showing a structural superposition of ORD-PI(4)P with the reported ORP3-ORD in apo-form. Since, they did not release the structure of the PI4P-bound form, we could not compare the structures of ligand-bound forms.

2. In addition, PI4P diC8 was used for crystallization trial (both yours and the reported complex), while the physiological PI4P molecules have longer acyl chains (C18/C20). Have you test the binding/extraction of BRAIN PI4P (and PIP2) by ORP3-ORD. Some sort of modelling would help.

>> Due to the solubility limitation of PI4P diC18, we used PI4P diC8 for crystallographic studies. Extraction of natural PI4P by ORP3 from the PM was recently reported (Elife 2020, PMID: 32234213). We could not obtain radioactive PI4P diC20 for a direct binding assay. Since ORP3 does not bind fluorescent sterols, it was not possible to test binding of PI4P diC18/C20 by sterol competition assays. In the revised manuscript, we included a structural model of PI4P diC20 in the binding pocket in Fig 3C.

3. Page 4, line 53-54, please add ORP2. Also please add the following reference for ORP5/8: PMID: 28970484. References 12 and 13 are for ORP1 and ORP2.

>> We have included the references in the text.

**Reviewer #2:**

The manuscript “Structure of human ORP3 ORD reveals conservation of a key function and ligand specificity in OSBP-related proteins” by Tong et al. presents a high-resolution structural model for human ORP3 and functional heterologous expression data that indicate an essential ORP3 function (and that other ORP family members as well, by inference) is PI4P dependent. The study does not identify the essential function. Nonetheless, the manuscript contributes useful, novel structure-function data to the ORP field in general. One particular strength of the manuscript is its comparative analysis of human and yeast ORP structures and structure-function analysis of human ORP3 in yeast, creating a not so common knowledge bridge between these experimental organisms.

Specific Remarks:

1. A key conclusion of the study is that PI4P binding to the ORP3 ORD is necessary for an essential function in yeast, based on the observation that the ORD K603E allele does not rescue the growth defect of osh delta osh4ts cells at restrictive temperature. Are wild-type ORD and ORD K603E expressed to the same level? If not, the conclusion cannot be made. Also, it is not clear in text or figure legends whether the constructs used in Fig. 4C are the same exact constructs as used in Fig. 4B.

>> In the revised manuscript, we have indicated that the constructs used in in the yeast complementation test and cell imaging were identical. The difference between two experiments was temperatures. The complementation test was performed at 37℃ and the yeast imaging was at 30℃. The yeast cells for imaging were grown at a permissive temperature to allow the growth of the yeast cells harboring the non-complementing ORP3 constructs. The expression level of ORD and ORD K603E at 30℃ were visualized in the figure 4B. Since the K603E mutant was made by single point mutagenesis of the wild type ORD, it is reasonable that the expression condition of both constructs are almost identical. The ORD K603E displays a cytosolic expression, which is similar to that of the ORD wild type.

2. Also in regard to Fig. 4B, the authors describe localization of ORP3 and its varying domains in the text (lines 263-276). The images presented do not support all the statements made in the text. For example, line 269, the authors claim that deletion of the PH domain (delta PH) eliminated the punctate localization at the PM. Yet, the image clearly shows punctate localization with the delta PH construct, albeit slightly more diffuse than with wild-type. Further on (line 270), the authors state that PH-ORD and delta FFAT show a membrane localization similar to that of the PH domain. I disagree; there is no similarity evident among the images presented.

>> Reviewer’s point is right. The localization of ΔPH is not completely cytosolic. We have modified the description in the revised version. The ΔPH constructs was basically cytosolic but also displayed punctate distribution which seems non-cortical ER localization, because the construct still contained an ER-targeting FFAT motif. The major effect of PH deletion was a loss of cortical ER-targeting due to the loss of a PM-binding domain. PH-ORD and ΔFFAT showed both cytosolic and PM distribution, whereas PH domain alone showed more intense PM targeting.

3. The Materials and Methods lack information, as follows.

a. Line 82. How was the secondary structure prediction made?

>> The secondary structures were predicted using PredictProtein server (http://predictprotein.org/).

b. Line 85 and 87. The authors report cell growth conditions in Kelvin rather than degree Celsius. Report in Celsius for ease of reading and keep to one scale throughout the manuscript.

>> We have changed Kevin to Celsius throughout the manuscript.

c. Line 92. Define the components of lysis buffer.

>> We have indicated 2X PBS buffer containing 20 mM imidazole as a lysis buffer.

d. Line 93. Was the elution with one buffer or two? If one, what was the pH of the buffer with

imidazole added?

>> Protein was eluted using one elution buffer. pH of the elution buffer containing 300 mM imidazole was adjusted to the final pH 8.0.

e. Line 94. How was the eluate concentrated?

>> We have added a description. The eluate was concentrated to 10 mg/ml using Amicon Ultra-15 centrifugal filter.

f. Line 95. How much thrombin was used?

>> The protein was cleaved with 10 international unit (IU) of thrombin protease per 10 mg of the recombinant protein.

g. Line 96. What was the wash and eluent for ion-exchange chromatography?

>> The ion exchange chromatography was performed using a linear gradient with buffer A composed of 50 mM MES-NaOH pH 6.5 and B buffer composed of 50 mM MES-NaOH pH 6.5, 500 mM NaCl.

h. Line 98. How was ORP3 ORD concentrated?

>> The eluate was concentrated to 10 mg/ml using Amicon Ultra-15 centrifugal filter (MWCO 10000).

i. Throughout the Materials and Methods, provide the source (supplier) of the reagents used,

especially less common reagents. Same for software applications.

>> We have included the suppliers for key reagents and software.

j. Line 105. Typo. Phosphoinositide.

>> We have fixed the typo.

k. Line 145. What type if site-directed mutagenesis?

>> Site-directed mutagenesis was performed using the protocol provided by QuickChange Site-Directed Mutagenesis kit (Agilent Technologies). We added the description in the revised manuscript.

l. Line 147. CBY926 genotype given as Osh1-7 delta: CEN osh4ts. This conveys, to some readers, that CEN osh4ts is linked to Osh1-7 delta. Recommend osh delta [CEN, osh4ts] or [pRS416MET25(osh4ts)]. Also, maintain consistency of nomenclature. Here, the strain is described as Osh1-7 delta; elsewhere in the manuscript it is described as osh

deleta.

>> We have changed the CBY926 genotype to *oshΔ*[CEN, osh4^ts^] throughout the manuscript as suggested.

m. Line 150. What was the dilution series? 10-fold dilutions? How long were plates incubated? What medium was used?

>> We have included the description in the text. The yeast cells were grown in a Hartwell's complete (HC) medium without uracil at a permissive temperature (30℃), and the five-fold dilution series were spotted on a HC medium agar plate and further incubated for 24h at 37℃.

n. Line 153. Is the pRS416 listed here the same as pRS416MET25 in line 145?

>> Yes. The plasmid vectors used for yeast complementation test and cell imaging are same. We have changed the name to pRS416MET25.

o. Line 155. Again, what medium was used?

>> The yeast cells were grown in a Hartwell's complete (HC) medium without uracil. We have indicated the medium in the text.

p. Line 155. Delete 1X. PBS is 1X unless specified differently.

>> We have deleted 1X.

q. Line 156 and elsewhere. “selective medium”, rather than “selection media”

>> We have modified to “a Hartwell's complete (HC) medium”.

r. Line 158. What was the exposure time (or range of times) for the image acquisition. Was any imaging processing performed? If yes, describe.

>> The green fluorescence of GFP-ORP3 constructs were imaged with 8 sec exposure. The images of yeast cells were cropped using the software ImageJ.

4. Lines 175-177. The authors reference Figs. 1C and 1D. It appears the text is best supported by Fig. 1B. The purpose of Fig. 1C is unclear. Fig. 1D is not necessary, but does provide useful data to readers.

>> The electron density of PI(4)P is very important in showing the quality of structural models particularly for the ligands, because ligand molecules are often not occupied 100% and not well-ordered. The recent report on the ORP3 structure (BBRC 529 (2020) 1005-1010) does not have high-resolution data for the ligand-bound form. The authors did not show the electron density of the ligand. They showed the ligand structure only in the paper and did not release the structural coordinates in the PDB. The conformation of the inositol ring of the PI(4)P shown in their paper seems inaccurate due to inversed modeling. I prefer to keep Fig 1C and Fig 1D, because they are informative to readers.

5. Line 284. The authors state that the PH domain and FFAT motif of human ORP3 was not essential for yeast growth. This is true because some yeast growth was observed when these constructs were expressed. However, the authors should state that only a partial rescue of the growth defect was observed, indicating that this domain and motif serve some function in yeast.

>> We have modified the description as the reviewer suggested.

6. Line 286. The authors refer to the K604E mutation, but Fig. 4 (all panels) indicate the mutation is K603E. Clarify.

>> K603E is the correct mutation. We have fixed the error.

7. Line 288. “defect” not “defeat”. Also Line 68.

>> We fixed the errors.

8. Throughout. “these data” not “this data”

>> We fixed the error.

9. Line 457. “Top view of Fig. 2A.” This is Fig. 2A. Do the authors mean to reference another figure?

>> The legends of Fig. 2A and 2B were switched by mistake. We fixed the error.

10. Line 471. The legend for Fig. 3D needs to be more descriptive.

>> We have included the detailed description of the figure in the legend as below. The shape of the ligand-binding pocket is shown as a transparent surface. The surface representation was sliced to visualize the interior of the ORP3 molecule. The outer and inner surfaces of ORP3 were shown in light grey and dark grey, respectively. The PI(4)P in the binding pocket is shown in a stick model. The cholesterol molecule was modeled to the pocket by superposing the structures of ORP3 and cholesterol-bound ORP1 (PDB id: 5ZM5). The cholesterol atoms clashing with the wall of the hydrophobic pocket are shown in light yellow.

11. Figure4C. PH-ORDKK60EE and DeltaFFAT-KK60EE not described in corresponding text. Describe data in figure.

>> We have added the description in the revision as follows. “Consistently, the KK60EE mutations in PH-ORD and ΔFFAT constructs abolished the PM localization but rescued the yeast growth defect.”
