# Supplementary material for: Structure of human ORP3 ORD reveals conservation of a key function and ligand specificity in OSBP-related proteins
Source: PLoS One. 2021 Apr 15;16(4):e0248781. doi: 10.1371/journal.pone.0248781 (PMC8049286; doi:10.1371/journal.pone.0248781)
Supplement: Attachment — Submitted filename: 2021_0225 Response to reviewers comments.docx [file pone.0248781.s004.docx]

**Response to the academic editor and reviewer’s comments**

**Academic editor:**

As was previously requested, there seems to be confusion about the necessity of wild-type transformation controls. The growth of CBY926 cells at 30˚C is not equivalent to wild type (there are obviously many additional genetic deletions involved). In addition, the expression of ORP3 does not have to cause lethality but can cause significant growth defects when expressed in wild-type yeast. Considering previous mistaken presumptions about the genetic nature of such ORP mutations, correctly providing proper controls is necessary for the clarity of such genetic experiments.

>> Previously, we misunderstood the editor's point. In the revision, we performed the complementation tests on the wild type yeast strain (CBY1) as suggested. Most of the ORP3 and OSBP1 constructs grew well. However, the constructs containing functional PH domain and ORD (PH-ORD and ΔFFAT) caused mild growth inhibition on the wild-type strain. We could not figure out the underlying inhibitory mechanism of the constructs on the wild-type strain. Perhaps, overexpression of human ORP3 PH-ORD lacking a FFAT motif might interfere with the normal function of yeast ORPs in PI4P metabolism. Still the main conclusion of the complementation test does not change.

**Reviewer #1**

My concerns have been addressed. This work is a great contribution to the study of this important family of proteins.

>> We appreciate the reviewer for the positive comments.

**Reviewer #2**

1. The authors have not satisfactorily addressed whether the levels of ORD and ORD K603E are the same. The authors, to support a key assertion in the manuscript, need to show a Western blot, rather than rely on fluorescence microscopy data because a several-fold difference in expression may be undetectable by non-quantitative fluorescence microscopy.

>> As the reviewer suggested, we performed Western blot analysis of the ORD wt and ORD K603E to check the expression levels. The expression of ORD K603E was 1.5 times higher than the ORD wt, confirming that non-complementation of ORD K603E is not caused by low expression but by the loss of function. We have included the procedure in the methods and added the results in the figure 4D.

2. The authors continue to over-interpret the fluorescence microscopy data in Fig 4B. In regard to the delta PH construct, the authors describe a punctate distribution "which seems non-cortical ER localization." This assignment of localization appears to be inferred by the presence of the FFAT motif rather than by any data within the figure. Recommend deleting "seems non-cortical ER localization."

>> As the reviewer suggested, we have deleted the description "seems non-cortical ER localization" In the revised manuscript. We have tone down the description and modified as “The ΔPH construct was mostly cytosolic but also displayed some punctate distribution inside the cells, possibly due to the presence of an ER-targeting FFAT motif.

With respect to PH-ORD and delta FFAT, the authors assert the localization is both cytosolic and PM, though less PM than with the PH domain alone. This could be true, but it is impossible to distinguish PM from cytosolic localization in the PH-ORD and delta FFAT images provided. The authors need to supply more convincing micrographs or retreat from this conclusion.

>> Due to the limitation of the accessible high-resolution microscopes, we could not provide new images with higher resolution. Instead, we have included the zoomed in images of the constructs with arrows indicating the PM localization in Fig 4B. We think the PM localization of PH-ORD and ΔFFAT can be identified in the current images. All of the PH, PH-ORD and ΔFFAT show similar bright GFP signals at the edges of the cells. However, the rim localization is lost when the PI4P-binding site in the PH domain is mutated in the PH-ORD (KK60-61EE), which suggests that the rim localization is mediated by the PM-targeting PH domain. The PH-ORD KK60EE shows the similar cytosolic localization which is observed for the ORD.
